# A high-precision genome size estimator based on the *k-mer* histogram correction

**DOI:** 10.3389/fgene.2024.1451730

**Published:** 2024-08-22

**Authors:** Xiangyu Liao, Wufei Zhu, Chaoyun Liu

**Affiliations:** ^1^ Department of Oncology, Yichang Central People’s Hospital, The First College of Clinical Medical Science, China Three Gorges University, Yichang, China; ^2^ Department of Endocrinology, Yichang Central People’s Hospital, The First College of Clinical Medical Science, China Three Gorges University, Yichang, China; ^3^ College of Information Engineering, Xi’an Mingde Institute of Technology, Xi’an, China

**Keywords:** next-generation sequencing, k-mer frequency distribution, k-mer histogram correction, genome size estimation, sequencing error, sequencing bias

## Abstract

**Introduction:**

In the realm of next-generation sequencing datasets, various characteristics can be extracted through *k-mer* based analysis. Among these characteristics, genome size (GS) is one that can be estimated with relative ease, yet achieving satisfactory accuracy, especially in the context of heterozygosity, remains a challenge.

**Methods:**

In this study, we introduce a high-precision genome size estimator, *GSET* (Genome Size Estimation Tool), which is based on *k-mer* histogram correction.

**Results:**

We have evaluated *GSET* on both simulated and real datasets. The experimental results demonstrate that this tool can estimate genome size with greater precision, even surpassing the accuracy of state-of-the-art tools. Notably, GSET also performs satisfactorily on heterozygous datasets, where other tools struggle to produce useable results.

**Discussion:**

The processing model of *GSET* diverges from the popular data fitting models used by similar tools. Instead, it is derived from empirical data and incorporates a correction term to mitigate the impact of sequencing errors on genome size estimation. *GSET* is freely available for use and can be accessed at the following URL: https://github.com/Xingyu-Liao/GSET.

## 1 Introduction

Estimating the size of a genome (GS) is a crucial step in understanding the intricacies of genome evolution and is often required in various aspects of genome sequencing and assembly (Sun et al., 2018). Experimental methods, such as feulgen densitometry and flow cytometry, offer one approach to this task. However, these methods can be complex due to their reliance on specialized instruments. In contrast, computational methods offer a different approach. These methods are based on the concept of *k-mers*, which are unique subsequences of a given length ‘k’ within the DNA sequence. By creating a histogram of these unique *k-mers* and applying mathematical models, these methods can provide a useful estimate of genome size. The initial model for this approach was designed for an ideal situation. It did not take into account factors such as repeat fragments and heterozygosity, and it assumed that the sequencing process was unbiased (Li and Waterman, 2003). However, real-world data often present more complicated situations. To address these complexities, several statistical distributions have been employed to fit the unique *k-mer* frequencies histogram. These include the Poisson distribution, the negative binomial distribution, and the skew normal distribution (Liu et al., 2012; Vurture et al., 2017). These distributions help to account for the variability and skewness that can occur in real sequencing data, thereby improving the accuracy of the genome size estimation. In conclusion, while experimental methods for genome size estimation have their place, the advent of *k-mer* based computational methods has provided a powerful tool for researchers. By continually refining these methods and models, we can hope to gain ever more accurate insights into the complex world of genomics.

Several methods have been proposed that utilize the fitting of the *k-mer* frequency histogram. For instance, *findGSE* (Guenzi-Tiberi et al., 2024) employs a skewed normal distribution to fit the *k-mer* frequency histogram. In contrast, *GenomeScope* (Oey et al., 2019; Thai et al., 2019) uses a mixture model of the negative binomial model for the same purpose. Both of these methods can effectively mitigate the estimation bias caused by sequencing errors and imbalances to a certain extent through fitting. However, their final estimation accuracy is contingent upon the degree of fit between the distribution characteristics of the actual sequencing data and its hypothetical distribution model. To elaborate, the *findGSE* method leverages the skewed normal distribution, which is a flexible model capable of capturing asymmetry in the *k-mer* frequency histogram. This allows it to handle a wide range of genome size estimation scenarios, including those with significant sequencing errors or imbalances. On the other hand, *GenomeScope* employs a mixture model of the negative binomial distribution. This model is particularly effective when dealing with overdispersed count data, which is common in genome size estimation tasks. The mixture model allows *GenomeScope* to capture the inherent variability in *k-mer* frequencies, leading to more accurate genome size estimates. However, it is important to note that while these methods can control the estimation bias to a certain extent, they are not foolproof. The accuracy of the final genome size estimate heavily depends on how well the chosen distribution fits the actual sequencing data. If the real data deviates significantly from the assumed distribution, the genome size estimate may be off. Therefore, it is crucial to choose the appropriate method based on the characteristics of the sequencing data at hand.

**FIGURE 1:**
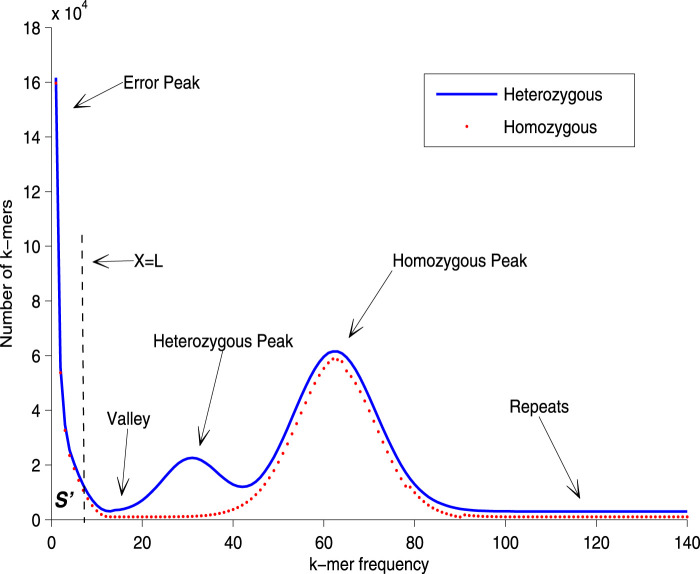
Unique k-mer histogram. In order to rectify the homozygous peak, the disturbance of the error peak must first be removed. The error rate of sequencing is utilized to estimate the number of error *k-mers*, $S^{\prime }$ , which are enclosed by the curve and the line X=L . All local maximum values within an interval size of 2L are then identified, and the position of the homozygous peak is determined by the highest of these local maximum values.

The unique *k-mer* distribution histogram, as depicted in Figure 1, provides a visual representation of the general cases of genome sequencing. The left peak of the curve primarily comprises *k-mers* that are a result of sequencing errors and biases, which occur at a low frequency. Conversely, the right tail of the curve is made up of repetitive fragments from the genome, which occur at a higher frequency. Recent models have employed various distributions to fit either the entire curve or a portion of it (Sun et al., 2018). The goal of these models is to closely align the fitted curve with the homozygous peak, thereby minimizing the impact of the error peak. This approach enhances the accuracy of genome size estimation. However, in the case of heterozygosity, a heterozygous peak appears between the valley and the homozygous peak. This additional peak complicates the fitting process, and the aforementioned distribution-based models may not perform well without modifications. In such scenarios, prior information can be invaluable in identifying the correct homozygous peak. In summary, while the unique *k-mer* distribution histogram and the associated mathematical models provide a powerful tool for genome size estimation, they also present challenges. These challenges, particularly in the case of heterozygosity, highlight the need for continual refinement of these models and the incorporation of prior information when available. By doing so, we can improve the accuracy and reliability of genome size estimation, thereby advancing our understanding of genome evolution.

In this study, we introduce a high-precision genome size estimator, *GSET* (Genome Size Estimation Tool), which leverages *k-mer* histogram correction. Unlike the popular data fitting models employed by similar tools, the processing model of *GSET* is derived from empirical data. It incorporates a correction term specifically designed to mitigate the impact of sequencing errors on genome size estimation. We have rigorously evaluated *GSET* using both simulated and real datasets. The experimental results demonstrate that *GSET* can estimate genome size with remarkable precision, even surpassing the accuracy of state-of-the-art tools currently available. Notably, *GSET* also performs exceptionally well on heterozygous datasets, a scenario where many other tools struggle to produce reliable results. This makes *GSET* a versatile and robust tool for genome size estimation across a variety of contexts.

## 2 Materials and methods

Assuming that the reference genome is a random sequence with no heterozygosity and no repeats, and the coverage of each position is uniformly distributed. Let N be the total number of *k-mers* in the sequencing data, G denote the size of the genome, and C represent the average depth of *k-mers*. A native genome size estimation model can be described as N=C∗(G−k+1) . As G≫k , we have G≈N/C , which is defined as the native model shown in Equation 1. To obtain the *k-mer* coverage (C) and the number of *k-mers* (N) , the homozygous peak in Figure 1 needs to be found. Thus, the total number of *k-mers* is $N=\sum_{1}^{n}x_{i}\times y_{i}$ , where $x_{i}$ is the frequency of the i -th *k-mer* and $y_{i}$ is the number of unique *k-mers* with frequency $x_{i}$ . The sum is over all unique *k-mers* in the sequencing data, and the formula for the genome size (G) based on the native model is:

$$G\approx \frac{N}{C}=\frac{\sum_{i=1}^{n}x_{i}\times y_{i}}{C}$$ (1)

Let $V\left(x_{v},y_{v}\right)$ and $M\left(x_{m},y_{m}\right)$ be the coordinates of the valley and homozygous peak, respectively. Ideally, the *k-mer* coverage C equals $C=x_{m}$ , and the genome size can then be estimated by $G\approx \left(\sum_{i=1}^{n}x_{i}\times y_{i}\right)/x_{m}$ . However, real sequencing datasets are more complicated due to biases in sequencing, sequencing errors, and repetitive fragments in the genome. Each of these situations can affect the accuracy of genome size estimation, and there is no precise pattern to describe these effects. Therefore, all of these factors are considered when finding correction factors from a large amount of datasets which have accurate references and known genome sizes. Statistical results show that most of the genome sizes (GS) estimated by the native model are larger than the real genome size. This is because the native model uses all *k-mers*, including error *k-mers*, to estimate the genome size. To refine the model, we set a dimensionless factor α , and use α×N/C to get the real GS . Statistical results show that $\alpha =x_{m}^{2}/\left(x_{v}^{2}+x_{m}^{2}\right)$ is the best correction factor. Therefore, the new genome size estimation model, GSET, can be expressed as follows.

$$G=\frac{x_{m}^{2}}{x_{v}^{2}+x_{m}^{2}}\times \frac{N}{C}=\frac{x_{m}\times \left(\sum_{i=1}^{n}x_{i}\times y_{i}\right)}{x_{v}^{2}+x_{m}^{2}}$$ (2)

**FIGURE 2:**
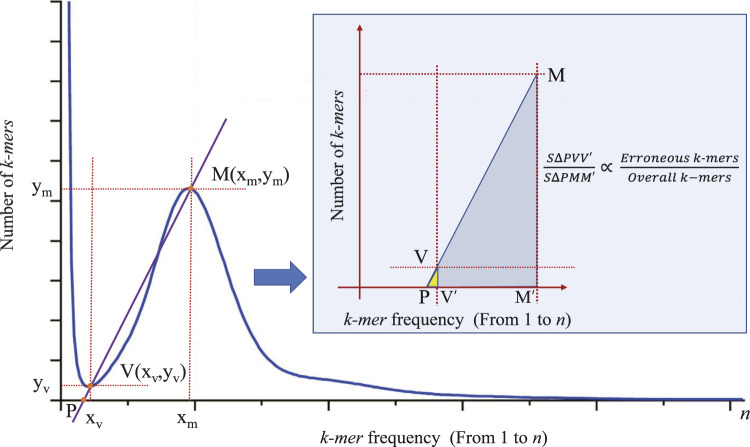
The principle of correcting the main peak of *k-mer* frequency distribution histogram. Points $V\left(x_{v},y_{v}\right)$ and $M\left(x_{m},y_{m}\right)$ represent the coordinates of the valley and homozygous peak, respectively. The line through points V ( $X_{v},Y_{v}$ ) and M ( $X_{m},Y_{m}$ ) intersects the X-axis at point P . Points $V^{\prime }\left(X_{v},0\right)$ and $M^{\prime }\left(X_{m},0\right)$ represent the perpendicular feet of points V and M on the X-axis, respectively. The ratio of the areas of $△PVV^{\prime }$ to $△0PMM^{\prime }$ is related to the number of erroneous *k-mers* relative to the overall *k-mer* count.

In the process of genome size estimation, when sequencing errors and bias occur, the corresponding histogram of the unique *k-mer* frequency may have an exponentially decreasing curve, just as the shown in the left part of Figure 1. These erroneous *k-mers* will often cause the main peak of the frequency distribution histogram to be shifted to the right compared to when there is no erroneous *k-mer*. In order to eliminate the negative impact of these erroneous *k-mers* on the accuracy of the main peak identification, we need to adopt certain strategies to correct the estimation (e.g., reducing the number of low-frequency *k-mer* participating in frequency statistics, that is, reducing the value of N ). In order to reduce the value of N , we multiply N by a correction coefficient α . The value of α ranges from 0 to 1. In practice, we found that the value of α is related to the number of erroneous *k-mer* and overall *k-mer*. In the context of the *k-mer* frequency histogram, the area enclosed by the curve and the X-axis represents the total number of *k-mers*. Consider the points V ( $X_{v},Y_{v}$ ) and M ( $X_{m},Y_{m}$ ) in Figure 2. The line passing through these points intersects the X-axis at point P . We observe that the area ratio of $△PVV^{\prime }$ to $△PMM^{\prime }$ correlates with the number of erroneous *k-mers* relative to the overall *k-mer* counts. Here, $V^{\prime }\left(X_{v},0\right)$ and $M^{\prime }\left(X_{m},0\right)$ represent the perpendicular feet of points V and M on the X-axis, respectively. Furthermore, we find that the square of the ratio $X_{v}$ to $X_{m}$ can represent the proportion between the number of erroneous *k-mers* and the total *k-mer* counts, as shown in Equation (2).

The rate and types of sequencing errors vary depending on the NGS platforms and library preparation methods. For instance, Illumina sequencing technologies exhibit error rates ranging from 0.5% to 2.5% (Kelley et al., 2010). These errors tend to occur more frequently in regions with extremely high GC or AT content, such as constant heterochromatin regions that include centromeres, telomeres, or highly repetitive sequences. While sequencing errors can impact the determination of X=L , they have minimal effect on identifying the homozygous peak. This is because, within the error tolerance range, we can ensure that there is no higher peak to the right of the X=L cutting line than the homozygous peak. The parameter L is influenced by the error rate of the input data. In Figure 1, when the error rate is denoted as α , the area of $S^{\prime }$ corresponds to α×S , where S represents the overall area enclosed by the curve and the coordinate axis. To find X=L , we start with x = 0 and gradually accumulate the area enclosed by the curve until it matches $S^{\prime }$ . At that point, the abscissa corresponds to X=L . The correction factor is specifically tied to the *k-mer* frequency histogram, simplifying its acquisition compared to methods relying on fitted distributions. Further details on identifying the valley and homozygous peak can be found in the Supplementary Material.

## 3 Results

We evaluated the performance of *GSET* on both four simulated datasets and nine real datasets. The simulated datasets were generated using *ART* (Huang et al., 2012), a widely used simulator. The real datasets ( R.sphaeroides , S.aureus , V.cholerae , M.abscessus , Human - chr14 , Saccharomyces , Melanogaster , Ant , and Mouse ) were downloaded from the GAGE-B (https://ccb.jhu.edu/gage_b/) and NCBI (https://www.ncbi.nlm.nih.gov/) websites. The first five datasets ( R.sphaeroides , S.aureus , V.cholerae , M.abscessus , Human - chr14 ) consist of HiSeq Illumina reads, while the latter datasets ( Saccharomyces , Melanogaster , Ant , and Mouse ) consist of MiSeq Illumina reads. A brief introduction of HiSeq and MiSeq technologies is as follows: 1) HiSeq is a high-throughput sequencing system developed by Illumina. It uses sequencing by synthesis (SBS) chemistry to generate large amounts of data with high accuracy. HiSeq systems, such as the HiSeq 2000 and HiSeq 2,500, are known for their flexibility, allowing for both rapid-run and high-output modes. They can produce up to 1 terabase (Tb) of data per run, making them suitable for large-scale genomic projects. 2) MiSeq is a benchtop sequencing system also developed by Illumina. It is designed for smaller-scale projects and offers a streamlined workflow from library preparation to data analysis. MiSeq uses the same SBS chemistry as HiSeq but is optimized for faster turnaround times and lower throughput. It can generate up to 15 gigabases (Gb) of data per run and is ideal for targeted gene sequencing, small genome sequencing, and 16S metagenomics. The genome size estimation results are summarized in Tables 1, 2 (Note: The lower the evaluation indicator 'accuracy', the better the result).

**TABLE 1:** The genome size estimated by different tools on simulated homozygous datasets.

Dataset	Ref. Len (bp)	GSET		findGSE		Genome scope		Native	
		Value (bp)	Accuracy	Value (bp)	Accuracy	Value (bp)	Accuracy	Value (bp)	Accuracy
SIM_dataset1	2,000,000	**1,999,164**	**0.004**	1,997,938	0.010	1,997,881	0.011	2,012,500	0.063
SIM_dataset2	2,040,000	**2,039,030**	**0.005**	2,041,272	0.006	2,036,239	0.018	2,090,851	0.249
SIM_dataset3	2,120,000	**2,119,321**	**0.003**	2,118,804	0.006	2,118,354	0.008	2,132,799	0.060
SIM_dataset4	2,140,000	2,139,623	0.002	2,141,695	0.008	**2,139,622**	**0.002**	2,171,586	0.148

*GSET*, *findGSE* and *GenomeScope* are three tools, and *Ref. Len* means the length of the reference genome. *Native* represents the genome size measured directly from the homozygous peak of the *k-mer* histogram without any correction. Datasets *SIM_dataset1* and *SIM_dataset4* are with the average read length of 125 bp, datasets *SIM_dataset2* and *SIM_dataset3* are with the average read length of 150 bp. Accuracy = $\;\;\frac{\left|The\;\;actual\;\;value\;\;of\;\;genome\;\;size\;-\;The\;\;estimation\;\;value\;\;of\;\;genome\;\;size\right|}{The\;\;actual\;\;value\;\;of\;\;genome\;\;size}$ . The simulated datasets are consisted of the paired-end reads. The values in bold provided in the table are the best results.

**TABLE 2:** The genome size estimated by different tools on real homozygous datasets.

Dataset	Ref. Len (bp)	GSET		findGSE		Genome scope		Native	
		Value (bp)	Accuracy	Value (bp)	Accuracy	Value (bp)	Accuracy	Value (bp)	Accuracy
R.sphaeroides	4,628,173	**4,693,604**	**0.014**	4,881,932	0.055	4,715,801	0.019	5,773,793	0.248
S.aureus	2,872,915	**2,867,262**	**0.002**	2,889,550	0.006	2,610,921	0.091	2,893,163	0.007
V.cholerae	4,033,464	4,048,812	0.004	**4,025,526**	**0.002**	3,826,668	0.051	4,370,906	0.084
M.abscessus	5,090,491	**4,936,399**	**0.030**	6,421,138	0.261	6,294,412	0.237	6,729,765	0.322
Human-chr14	107,349,540	**99,169,509**	**0.076**	91,798,688	0.145	88,076,272	0.180	93,336,008	0.131
Ant	295,944,863	321,157,729	0.033	**296,098,286**	**0.005**	295,025,758	0.003	335,121,109	0.132
Mouse	2,818,974,548	**2,823,422,104**	**0.002**	2,785,210,329	0.011	2,745,274,960	0.026	3,336,771,577	0.184
Melanogaster	168,736,537	**168,861,701**	**0.001**	146,044,559	0.134	139,401,686	0.174	183,545,327	0.088
Saccharomyces	12,111,892	**12,355,209**	**0.020**	14,585,622	0.204	14,711,912	0.215	14,120,239	0.166

*GSET*, *findGSE* and *GenomeScope* are three tools, and *Ref. Len* means the length of the reference genome. *Native* represents the genome size measured directly from the homozygous peak of the *k-mer* histogram without any correction. The read file size of the Mouse dataset exceeds 150GB, and the corresponding genome length is close to 3 GB. Accuracy = $\;\;\frac{\left|The\;\;actual\;\;value\;\;of\;\;genome\;\;size\;-\;The\;\;estimation\;\;value\;\;of\;\;genome\;\;size\right|}{The\;\;actual\;\;value\;\;of\;\;genome\;\;size}$ . The real datasets are consisted of the paired-end reads. The values in bold provided in the table are the best results.

Compared to three other methods, *GSET* demonstrated superior accuracy. We further evaluated *GSET*’s performance on heterozygous datasets. Our experiments involved both simulated datasets generated by PIRS (Hu et al., 2012) and a real dataset (C.elegans) obtained from the Bioinformation and DDBJ Center (https://www.ddbj.nig.ac.jp/index-e.html). The heterozygosity rate for this dataset is 0.5 (Kajitani et al., 2014). Detailed results can be found in Supplementary Tables S1–S9 of the Supplementary Material. Notably, in heterozygous cases, the accuracy of various methods tends to be lower due to the interference from heterozygous peaks. In order to verify the performance of *GSET* on large real datasets, we conducted experiments using NGS datasets from species such as Saccharomyces , Melanogaster , Ant , and Mouse . Notably, the read file size of the Mouse dataset exceeds 150GB, with a corresponding genome length close to 3 GB (paired-end read files ERR2894257, ERR2894259, and ERR2894260 were downloaded from NCBI). The test results, as shown in Table 2, demonstrate that *GSET* achieved the highest estimation accuracy for datasets including Mouse , Melanogaster , and Saccharomyces .

**FIGURE 3:**
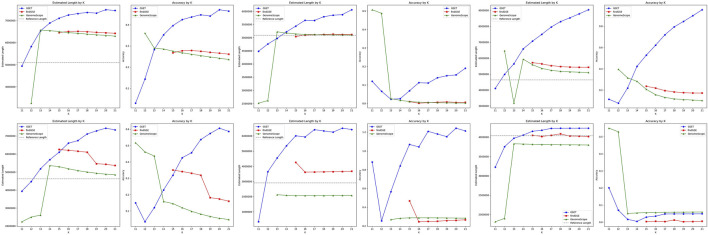
Performance comparison of genome size estimation using three tools: *GSET*, *findGSE*, and *GenomeScope* on real homozygous datasets.

The performance comparison of GSET , findGSE , and GenomeScope for genome size estimation on real homozygous datasets are shown in Figure 3. The estimated length of GSET consistently increases with the increment of k across all datasets, reaching its maximum estimated length and accuracy at k = 21 in most datasets. This suggests a robust adaptation of GSET to genomic complexities, significantly outperforming findGSE and GenomeScope in these scenarios. For findGSE , performance in terms of estimated length and accuracy decreases as k increases across all datasets, indicating potential limitations in resolving more complex genomic structures within a narrow range of k values. On the other hand, GenomeScope achieves peak performance in terms of both estimated length and accuracy at k = 13 for most datasets, with a declining trend observed as k increases. This highlights its optimized performance at lower k values but suggests a decrease in effectiveness in accurately capturing genomic size as k increases.

**FIGURE 4:**
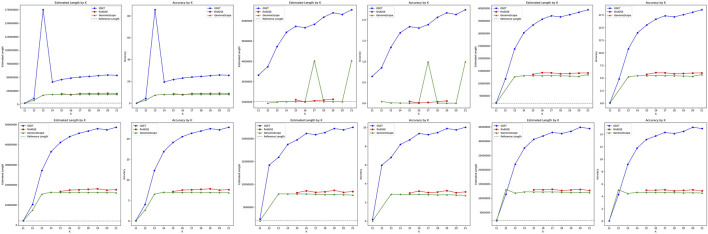
Performance comparison of genome size estimation using three tools: *GSET*, *findGSE*, and *GenomeScope* on the simulated heterozygous datasets.

The performance comparison of GSET , findGSE , and GenomeScope for genome size estimation on simulated heterozygous datasets are shown in Figure 4. GSET consistently achieved the highest values in estimated length and accuracy across all datasets, significantly surpassing findGSE and GenomeScope . This indicates GSET ?s superior capability in handling heterozygous genomic complexities. As k increases, both the estimated length and accuracy of GSET also show an upward trend. However, in the test2020000.300.75-0.02–0.05 dataset, GSET reached its peak performance at k = 13, with significantly lower performance at other k values. This suggests a potential optimal k setting for this specific type of genomic data within GSET ’s algorithm. On the other hand, findGSE and GenomeScope generally exhibit similar performance, with findGSE marginally outperforming GenomeScope . Despite this, both tools are significantly inferior to GSET , indicating their less effective handling of heterozygous genomes. Furthermore, the performance of findGSE and GenomeScope becomes less sensitive to changes in k beyond k = 12, suggesting a plateau in their capability to further resolve genomic details at higher k values.

## 4 Discussion

In this study, we address specific principles, delve into implementation details, and engage with user concerns. We meticulously respond to these issues in a systematic manner across the subsequent sections.

### 4.1 Novelty of the proposed method

The methods for estimating genome size typically fall into two categories: experimental methods and estimation methods. Experimental techniques, such as flow cytometry and Southern blotting, are costly and require specialized equipment. Estimation methods, on the other hand, can be broadly classified based on their approach. The first category involves analyzing the *k-mer* frequency distribution histogram, while the second relies on sequence alignment and assembly. Both estimation methods utilize the original sequencing reads, but the latter demands more computational resources. Among the tools commonly used for *k-mer*-based estimation, three stand out: *findGSE*, *GenomeScope*, and our proposed method, *GSET*. While *findGSE* fits the *k-mer* frequency histogram using a skewed normal distribution, *GenomeScope* employs a mixture model based on the negative binomial distribution. These approaches effectively mitigate estimation bias caused by sequencing errors and imbalances, but their accuracy hinges on how well their distribution models match the real sequencing data. In contrast, our method directly analyzes the original *k-mer* frequency distribution, offering three key advantages. First, it ensures the integrity of the information used. Second, it achieves a zero-deviation fit with the actual distribution. Finally, correction coefficients reduce the impact of erroneous *k-mers*, enhancing estimation accuracy. Experimental results demonstrate that our approach outperforms the former two methods.

### 4.2 Selection of the *k-mer* size in estimation

Selecting the appropriate value of (k) significantly impacts the accuracy of genome size estimation, regardless of whether we use (findGSE) , (GenomeScope) , or our proposed method, (GSET) . For instance, (findGSE) provides a range of (k) values (from 15 bp to 21 bp) but does not prescribe a specific method for determining (k) . On the other hand, (GenomeScope) not only lacks a predefined range for (k) , but it also requires users to manually specify the exact (k) value. Consequently, none of the existing tools offers a flawless solution to this challenge. In our study, we propose solutions for two scenarios. First, if we know the approximate genome size of a related species or have an estimate for the rough genome size, we can determine the specific value of (k) using Equation (3). Here, (k) represents the *k-mer* size, and (G) corresponds to the approximate size of either the rough genome or the genome of a similar species (Price et al., 2005).

$$k=\left\lceil \log_{4}G+1\right\rceil$$ (3)

Additionally, if the species is unknown, we recommend observing the *k-mer* frequency histogram to assess whether the current value of k is appropriate. When the value of k is suitable, the waveform of the *k-mer* frequency histogram closely resembles that shown in Figure 1. Conversely, if the valley corresponding to $X_{v}$ (minimum frequency) and the peak corresponding to $X_{m}$ (maximum frequency) are not distinct, with no significant difference in height, it indicates that the chosen value of k is not appropriate.

### 4.3 Application scenarios of *GSET*

**FIGURE 5:**
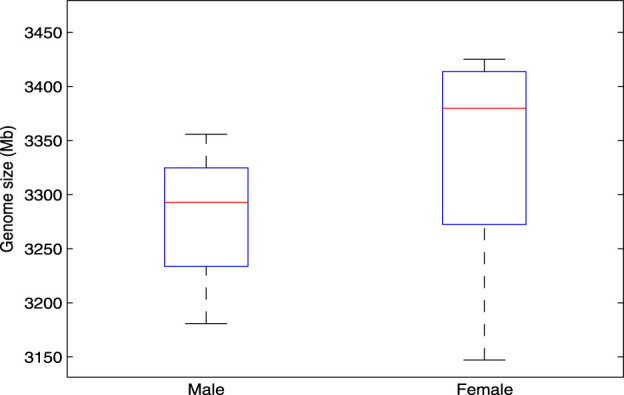
A stable difference in genome length exists between male and female genomes, as estimated by the GSET tool. In this study, we investigated stable differences in genome length between male and female individuals using the GSET Tool. The average genome size for males was estimated to be 3,279 Mb, while females had an average genome size of 3,428 Mb. These findings align with the known chromosomal differences: females possess two X chromosomes, whereas males have one X and one Y chromosome.

In the research of bioinformatics, genome size estimation has been applied in many aspects (Bosco et al., 2007; Gao et al., 2014). For example, in the applications of species classification and evolutionary relationship analysis, by estimating the genome size of the sequenced samples, we can quickly cluster these samples and infer the evolutionary relationship among them. In this study, we consider using GSET to analyze the stable differences between male and female genomes. For example, we downloaded 14 human sequencing datasets on the NCBI website, of which 7 are males and the remaining 7 are females (Paired-end reads files ERR1347682, ERR1347702, ERR1347706, ERR1347728, ERR1347738, ERR1395547, ERR1395570, ERR1347657, ERR1347661, ERR1347662, ERR1347672, ERR1347679, ERR1347707 and ERR1419089 are downloaded from NCBI https://www.ncbi.nlm.nih.gov/sra/). The genome sizes of these 14 samples are all estimated by GSET when k is set to 21 bp. The average genome sizes of male and female in the experiment are 3,279 Mb and 3,428 Mb, respectively. This conclusion is consistent with the fact: The female genome contains two X chromosomes, and male genome contains one X chromosome and one Y chromosome. Therefore, the female genome size is a bit bigger than that of male. The detailed experimental results are shown in Figure 5.

## 5 Conclusion

In this paper, we present *GSET* to realize an higher accuracy genome size estimation, the mathematical model of *GSET* is concise, and is easily to be computed than the fitting based methods. Through the correction factor, we get a satisfactory estimation results on both simulated datasets and real datasets, even on the situation of heterozygous, it can also give out more useable results than other distribution based methods. *GSET* could be used for analysis which need a relatively higher accuracy estimation of genome size in the next-generation sequencing data.

## Data Availability

The original contributions presented in the study are included in the article/Supplementary Material, further inquiries can be directed to the corresponding author.

## References

[B1] Bosco G. Campbell P. Leiva-Neto J. T. Markow T. A. (2007). Analysis of drosophila species genome size and satellite dna content reveals significant differences among strains as well as between species. Genetics 177 (3), 1277–1290. 10.1534/genetics.107.075069 18039867 PMC2147996

[B2] Gao X.-Y. Zhi X.-Y. Li H.-W. Klenk H. P. Li W. J. (2014). Comparative genomics of the bacterial genus streptococcus illuminates evolutionary implications of species groups. PLoS ONE 9 (6), 101229. 10.1371/journal.pone.0101229 PMC4076318 24977706

[B3] Guenzi-Tiberi P. Istace B. Alsos I. G. Coissac E. Lavergne S. Aury J. M. (2024). Locogse, a sequence-based genome size estimator for plants. Front. Plant Sci. 15, 1328966. 10.3389/fpls.2024.1328966 38550287 PMC10972871

[B4] Hu X. Yuan J. Shi Y. Lu J. Liu B. Li Z. (2012). pirs: profile-based illumina pair-end reads simulator. Bioinformatics 28 (11), 1533–1535. 10.1093/bioinformatics/bts187 22508794

[B5] Huang W. Li L. Myers J. R. Marth G. T. (2012). Art: a next-generation sequencing read simulator. Bioinformatics 28 (4), 593–594. 10.1093/bioinformatics/btr708 22199392 PMC3278762

[B6] Kajitani R. Toshimoto K. Noguchi H. Toyoda A. Ogura Y. Okuno M. (2014). Efficient *de novo* assembly of highly heterozygous genomes from whole-genome shotgun short reads. Genome Res. 24 (8), 1384–1395. 10.1101/gr.170720.113 24755901 PMC4120091

[B7] Kelley D. R. Schatz M. C. Salzberg S. L. (2010). Quake: quality-aware detection and correction of sequencing errors. Genome Biol. 11, 116. 10.1186/gb-2010-11-11-r116 21114842 PMC3156955

[B8] Li X. Waterman M. S. (2003). Estimating the repeat structure and length of dna sequences using l-tuples. Genome Res. 13 (8), 1916–1922. 10.1101/gr.1251803 12902383 PMC403783

[B9] Liu B. Shi Y. Yuan J. Hu X. Zhang H. Li N. (2012). Estimation of genomic characteristics by analyzing k-mer frequency in *de novo* genome projects. arXiv, 1308. 10.48550/arXiv.1308.2012

[B10] Oey H. Zakrzewski M. Gravermann K. Young N. D. Korhonen P. K. Gobert G. N. (2019). Whole-genome sequence of the bovine blood fluke schistosoma bovis supports interspecific hybridization with s. haematobium. PLOS Pathog. 15, 1007513. 10.1371/journal.ppat.1007513 PMC6361461 30673782

[B11] Price A. L. Jones N. C. Pevzner P. A. (2005). *De novo* identification of repeat families in large genomes. Genome Res. 21, 351–358. 10.1093/bioinformatics/bti1018 15961478

[B12] Sun H. Ding J. Piednoël M. Schneeberger K. (2018). findgse: estimating genome size variation within human and arabidopsis using k-mer frequencies. Bioinformatics 4 (34), 550–557. 10.1093/bioinformatics/btx637 29444236

[B13] Thai B. T. Lee Y. P. Gan H. M. Austin C. M. Croft L. J. Trieu T. A. (2019). Whole genome assembly of the snout otter clam, lutraria rhynchaena, using nanopore and illumina data, benchmarked against bivalve genome assemblies. Front. Genet. 10, 1158. 10.3389/fgene.2019.01158 31824566 PMC6880199

[B14] Vurture G. W. Sedlazeck F. J. Nattestad M. Underwood C. J. Fang H. Gurtowski J. (2017). Genomescope: fast reference-free genome profiling from short reads. Bioinformatics 33 (14), 2202–2204. 10.1093/bioinformatics/btx153 28369201 PMC5870704

