# Supplementary material for: A high-precision genome size estimator based on the k-mer histogram correction
Source: Front Genet. 2024 Aug 22;15:1451730. doi: 10.3389/fgene.2024.1451730 (PMC11374637; doi:10.3389/fgene.2024.1451730)
Supplement: Supplementary file 1 [file DataSheet1.PDF]

Supplemental Results

of

A high-precision genome size estimator based on the *k-mer* histogram correction

Xiangyu Liao [1], Wufei Zhu[2] and Chaoyun Liu [3,*]

[1]Department of Oncology, Yichang Central People's Hospital, The First College of Clinical Medical Science, China Three Gorges University, Yichang 443000, P.R. China..

[2]Department of Endocrinology, Yichang Central People's Hospital, The First College of Clinical Medical Science, China Three Gorges University, Yichang 443000, P.R. China..

[3] College of Information Engineering, Xi'an Mingde Institute of Technology, Xi'an 710000, P.R. China.

* Corresponding author: Chaoyun Liu (superchaoyun@126.com)

1. Method for finding valley and homozygous peak in unqiue k-mer histogram

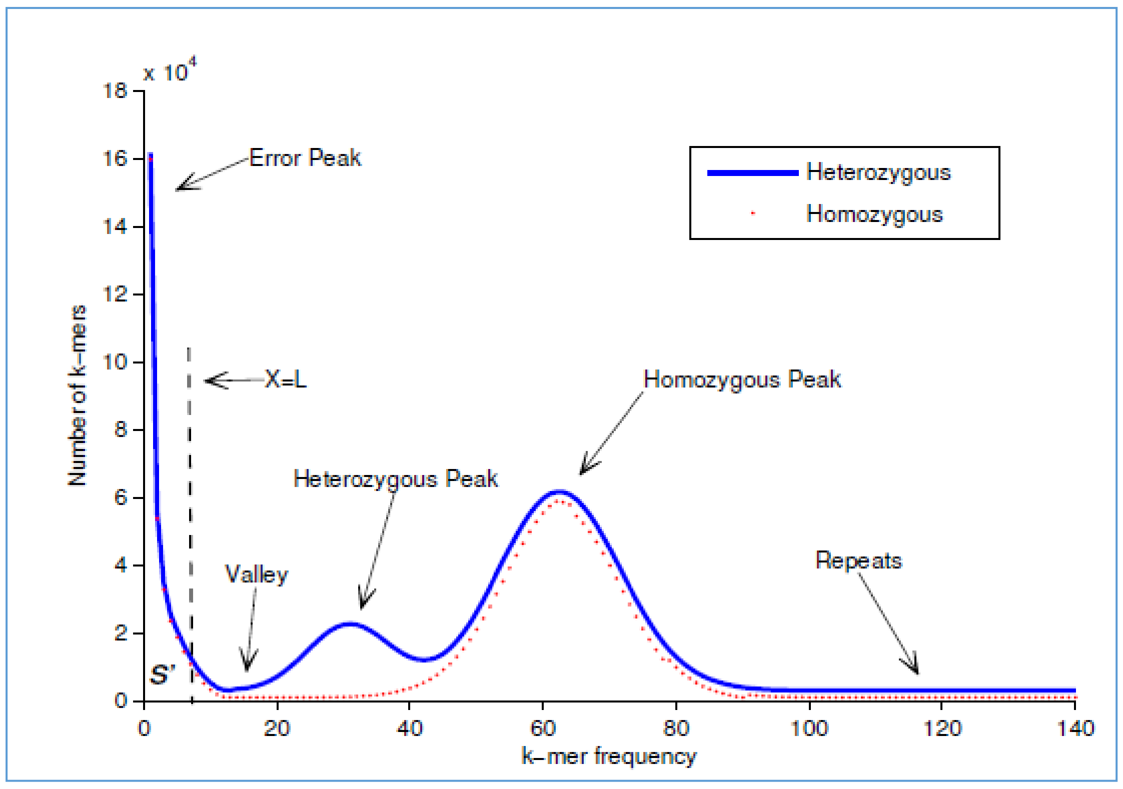

Fig. S1 Unique *k-mer* histogram

The details of finding valley and homozygous peak:

The key step of the proposed method involves finding the homozygous peak and the valley of the curve (Fig. S1). To identify the homozygous peak, we first need to eliminate the disturbance caused by the error peak. We use the error rate of NGS sequencing to estimate the number of error *k-mers*, *S'*, which are enclosed by the curve and the cutting line $X = L$. Subsequently, we find all the local maximum values within an interval size of $2L$. The position of the homozygous peak is determined by the highest peak among these local maximum values. The valley is identified by the minimum value between $X=L$ and the homozygous peak.

2. Settings of tools used in comparison

(1) *Jellyfish* is used to get the k-mer histogram file of reads, command line like:
zcat *.fq.gz | jellyfish count /dev/fd/0 -C -o test_15mer -m 15 -t 8 -s 1G
jellyfish histo -h 3000000 -o test_15mer.histo test_15mer

(2.1) *GSE* is used to estimate the reference length in Matlab, run the command:
GSE('test_15mer.histo', 99, 15 , 0.05);
In this command, test_15mer.histo is the name of the input file, 99 is the read length, and k=15, error rate=0.05.

(2.2) *findGSE* is used to estimate the reference length in R:
library("findGSE")
findGSE(histo="test_15mer.histo", sizek=15, outdir="outdir")

(2.3) *GenomeScope* is used to estimate the reference length in R:
Rscript genomescope.R test_15mer.histo 15 99 outdir

3. Commands for generating simulated datasets

Pirs is used to generate the simulated homozygous datasets, the command line is:
pirs simulate -i random_sequences_set.fa -m 250 -l 99 -x 50 -v 10 -e 0.01
the insertsize is 250, the read length is 99, the read coverage is 50, the standard deviation of insert sizes is 20, and the error rate is 0.01, the reads are derived from the input fasta file random_sequences_set.fa

ART is used to generate the simulated homozygous datasets, the command line is:
art_illumina -ss HS25 -i test2000000.fa -p -l 125 -f 70 -m 200 -s 10 -o test2000000_125_70_
HS25 means the name of Illumina sequencing system is HiSeq 2500, the reads are derived from the input fasta file test2000000.fa, and the read length is 125, the read coverage is 70, the mean size of DNA/RNA fragments for paired-end simulations is 200, and the standard deviation of DNA/RNA fragment size is 10, the prefix of output filename is test2000000_125_70_
Pirs is used to generate the simulated heterozygous datasets, the command line should add the **–I** parameter, which infers the other reference genome sequence that make a heterozygous part of all reads.

4. Detailed Experimental Results

1) Experimental results of three different tools on homologous and heterozygous datasets

Tables S1 to S3 show the effects of genome size estimation on the real homozygous datasets by the three tools.
Tables S4 to S6 show the effects of genome size estimation on the simulated heterozygous datasets by the three tools.
Tables S7 to S9 show the effects of genome size estimation on the real heterozygous datasets by the three tools.

Field label instruction:

* ***Ref_Length*** : The length of the reference genome.
* ***Error_rate*** : The error rate of sequencing.
* ***K*** : The k-mer size.
* ***L*** : The x-axis coordinate corresponding to the right boundary of the removed region of the k-mer histogram.
* ***Est.RefLen*** : The length of reference genome which estimated by the tool.
* ***Accurancy*** : This is an index that reflects the accuracy of estimation. $\boldsymbol{Accurancy} = \frac{|\textit{The actual value of genome size} - \textit{The estimated value of genome size}|}{\textit{The actual value of genome size}}$

Table S1. Genome size estimation on real homozygous datesets generated by Pirs using GEST

DataSet	Ref_Length	Error_rate	K	L	Est.RefLen	Accurancy
A_hydrophila_HiSeq	4673830	0.005	11	17	3897767.032	0.16604
A_hydrophila_HiSeq	4673830	0.005	12	4	4183349.287	0.10494
A_hydrophila_HiSeq	4673830	0.005	13	1	4294113.693	0.081243
A_hydrophila_HiSeq	4673830	0.005	14	1	4445345.363	0.048886
A_hydrophila_HiSeq	4673830	0.005	15	1	4616182.304	0.012334
A_hydrophila_HiSeq	4673830	0.005	16	1	4791851.914	0.025252
A_hydrophila_HiSeq	4673830	0.005	17	1	4809706.093	0.029072
A_hydrophila_HiSeq	4673830	0.005	18	1	4792772.329	0.025449
A_hydrophila_HiSeq	4673830	0.005	19	1	4793230.639	0.025547
A_hydrophila_HiSeq	4673830	0.005	20	1	4835003.979	0.034484
A_hydrophila_HiSeq	4673830	0.005	21	1	4817367.993	0.030711
B_fragilis_HiSeq	5373121	0.005	11	33	4966321.311	0.07571
B_fragilis_HiSeq	5373121	0.005	12	6	4745926.753	0.11673
B_fragilis_HiSeq	5373121	0.005	13	1	5289462.919	0.01557
B_fragilis_HiSeq	5373121	0.005	14	1	5344674.08	0.0052943
B_fragilis_HiSeq	5373121	0.005	15	1	5370382.035	0.00050975
B_fragilis_HiSeq	5373121	0.005	16	1	5391342.399	0.0033912
B_fragilis_HiSeq	5373121	0.005	17	1	5436862.017	0.011863
B_fragilis_HiSeq	5373121	0.005	18	1	5469227.99	0.017887
B_fragilis_HiSeq	5373121	0.005	19	1	5595687.791	0.041422
B_fragilis_HiSeq	5373121	0.005	20	1	5581065.089	0.038701
B_fragilis_HiSeq	5373121	0.005	21	1	5539407.714	0.030948
Human_Chromosome_14	107349540	0.005	11	114	2857664.202	0.97338
Human_Chromosome_14	107349540	0.005	12	30	1295035.407	0.98794
Human_Chromosome_14	107349540	0.005	13	4	69703729.56	0.35068
Human_Chromosome_14	107349540	0.005	14	1	80639574.66	0.24881
Human_Chromosome_14	107349540	0.005	15	1	88593503.41	0.17472
Human_Chromosome_14	107349540	0.005	16	1	93219126.09	0.13163
Human_Chromosome_14	107349540	0.005	17	1	97403098.85	0.092655
Human_Chromosome_14	107349540	0.005	18	1	98059415.87	0.086541
Human_Chromosome_14	107349540	0.005	19	1	96886158.93	0.09747
Human_Chromosome_14	107349540	0.005	20	1	98624173.19	0.08128
Human_Chromosome_14	107349540	0.005	21	1	99169508.67	0.0762
M_abscessus_HiSeq	5090491	0.005	11	11	4483609.593	0.11922
M_abscessus_HiSeq	5090491	0.005	12	2	4756374.95	0.065635
M_abscessus_HiSeq	5090491	0.005	13	1	4974151.847	0.022854
M_abscessus_HiSeq	5090491	0.005	14	1	5219616.559	0.025366
M_abscessus_HiSeq	5090491	0.005	15	1	5437917.056	0.06825
M_abscessus_HiSeq	5090491	0.005	16	1	5660229.197	0.11192
M_abscessus_HiSeq	5090491	0.005	17	1	5652535.406	0.11041
M_abscessus_HiSeq	5090491	0.005	18	1	5796452.853	0.13868
M_abscessus_HiSeq	5090491	0.005	19	1	5851897.218	0.14957
M_abscessus_HiSeq	5090491	0.005	20	1	5874824.653	0.15408
M_abscessus_HiSeq	5090491	0.005	21	1	6059986.567	0.19045
M_abscessus_MiSeq	5090491	0.005	11	11	4936398.946	0.030271
M_abscessus_MiSeq	5090491	0.005	12	2	5823103.74	0.14392

M_abscessus_MiSeq	5090491	0.005	13	1	6533066.663	0.28339
M_abscessus_MiSeq	5090491	0.005	14	1	6886948.465	0.3529
M_abscessus_MiSeq	5090491	0.005	15	1	7107808.039	0.39629
M_abscessus_MiSeq	5090491	0.005	16	1	7250687.077	0.42436
M_abscessus_MiSeq	5090491	0.005	17	1	7313980.698	0.43679
M_abscessus_MiSeq	5090491	0.005	18	1	7367642.281	0.44733
M_abscessus_MiSeq	5090491	0.005	19	1	7336076.746	0.44113
M_abscessus_MiSeq	5090491	0.005	20	1	7488910.059	0.47116
M_abscessus_MiSeq	5090491	0.005	21	1	7456554.808	0.4648
R_sphaeroides_HiSeq	4628173	0.005	11	14	3522767.269	0.23884
R_sphaeroides_HiSeq	4628173	0.005	12	3	4179558.851	0.096931
R_sphaeroides_HiSeq	4628173	0.005	13	1	4693604.863	0.014138
R_sphaeroides_HiSeq	4628173	0.005	14	1	5226266.586	0.12923
R_sphaeroides_HiSeq	4628173	0.005	15	1	5570731.463	0.20366
R_sphaeroides_HiSeq	4628173	0.005	16	1	5694463.586	0.23039
R_sphaeroides_HiSeq	4628173	0.005	17	1	6138364.062	0.3263
R_sphaeroides_HiSeq	4628173	0.005	18	1	6066107.227	0.31069
R_sphaeroides_HiSeq	4628173	0.005	19	1	6356285.501	0.37339
R_sphaeroides_HiSeq	4628173	0.005	20	1	6532879.321	0.41155
R_sphaeroides_HiSeq	4628173	0.005	21	1	6684802.186	0.44437
R_sphaeroides_MiSeq	4628173	0.005	11	10	4100337.852	0.11405
R_sphaeroides_MiSeq	4628173	0.005	12	2	4985732.342	0.077257
R_sphaeroides_MiSeq	4628173	0.005	13	1	5645380.976	0.21979
R_sphaeroides_MiSeq	4628173	0.005	14	1	6583555.87	0.4225
R_sphaeroides_MiSeq	4628173	0.005	15	1	7066821.14	0.52691
R_sphaeroides_MiSeq	4628173	0.005	16	1	7500933.296	0.62071
R_sphaeroides_MiSeq	4628173	0.005	17	1	7953298.199	0.71845
R_sphaeroides_MiSeq	4628173	0.005	18	1	8297600.954	0.79285
R_sphaeroides_MiSeq	4628173	0.005	19	1	8533273.102	0.84377
R_sphaeroides_MiSeq	4628173	0.005	20	1	8781269.421	0.89735
R_sphaeroides_MiSeq	4628173	0.005	21	1	9042277.915	0.95375
Rhodobacter_sphaeroides	4628173	0.005	11	3	3938488.175	0.14902
Rhodobacter_sphaeroides	4628173	0.005	12	1	4471386.248	0.033877
Rhodobacter_sphaeroides	4628173	0.005	13	1	5182507.886	0.11977
Rhodobacter_sphaeroides	4628173	0.005	14	1	5685446.92	0.22844
Rhodobacter_sphaeroides	4628173	0.005	15	1	6102739.145	0.31861
Rhodobacter_sphaeroides	4628173	0.005	16	1	6598402.74	0.4257
Rhodobacter_sphaeroides	4628173	0.005	17	1	6739470.421	0.45618
Rhodobacter_sphaeroides	4628173	0.005	18	1	7111260.273	0.53652
Rhodobacter_sphaeroides	4628173	0.005	19	1	7281740.517	0.57335
Rhodobacter_sphaeroides	4628173	0.005	20	1	7432705.746	0.60597
Rhodobacter_sphaeroides	4628173	0.005	21	1	7340419.688	0.58603
S_aureus_HiSeq	2872915	0.005	11	10	2359799.001	0.1786
S_aureus_HiSeq	2872915	0.005	12	3	2639927.818	0.081098
S_aureus_HiSeq	2872915	0.005	13	1	2934723.445	0.021514
S_aureus_HiSeq	2872915	0.005	14	1	2853373.699	0.0068019
S_aureus_HiSeq	2872915	0.005	15	1	2867262.964	0.0019674
S_aureus_HiSeq	2872915	0.005	16	1	2891376.03	0.0064259
S_aureus_HiSeq	2872915	0.005	17	1	3003128.062	0.045324
S_aureus_HiSeq	2872915	0.005	18	1	2993817.169	0.042083
S_aureus_HiSeq	2872915	0.005	19	1	3013434.901	0.048912
S_aureus_HiSeq	2872915	0.005	20	1	3036008.611	0.056769
S_aureus_HiSeq	2872915	0.005	21	1	3040630.953	0.058378
Staphylococcus_aureus	2903107	0.005	11	4	342718.1911	0.88195
Staphylococcus_aureus	2903107	0.005	12	1	3640464.832	0.25399
Staphylococcus_aureus	2903107	0.005	13	1	4544989.24	0.56556
Staphylococcus_aureus	2903107	0.005	14	1	5343982.314	0.84078
Staphylococcus_aureus	2903107	0.005	15	1	6004046.575	1.0681
Staphylococcus_aureus	2903107	0.005	16	1	5928501.096	1.0421
Staphylococcus_aureus	2903107	0.005	17	1	6409297.92	1.2077
Staphylococcus_aureus	2903107	0.005	18	1	6327117.36	1.1794
Staphylococcus_aureus	2903107	0.005	19	1	6245122.26	1.1512
Staphylococcus_aureus	2903107	0.005	20	1	6507537.584	1.2416
Staphylococcus_aureus	2903107	0.005	21	1	6421642.072	1.212
V_cholerae_HiSeq	4033464	0.005	11	9	3224261.326	0.20062
V_cholerae_HiSeq	4033464	0.005	12	2	3751317.49	0.069951
V_cholerae_HiSeq	4033464	0.005	13	1	3967902.325	0.016254
V_cholerae_HiSeq	4033464	0.005	14	1	4048812.801	0.0038054
V_cholerae_HiSeq	4033464	0.005	15	1	4149918.547	0.028872
V_cholerae_HiSeq	4033464	0.005	16	1	4173805.342	0.034794

V_cholerae_HiSeq	4033464	0.005	17	1	4225070.729	0.047504
V_cholerae_HiSeq	4033464	0.005	18	1	4226044.741	0.047746
V_cholerae_HiSeq	4033464	0.005	19	1	4226961.779	0.047973
V_cholerae_HiSeq	4033464	0.005	20	1	4227820.789	0.048186
V_cholerae_HiSeq	4033464	0.005	21	1	4228614.716	0.048383
V_cholerae_MiSeq	4033464	0.005	11	9	3914283.927	0.029548
V_cholerae_MiSeq	4033464	0.005	12	2	4479103.049	0.11049
V_cholerae_MiSeq	4033464	0.005	13	1	4717339.617	0.16955
V_cholerae_MiSeq	4033464	0.005	14	1	5060376.441	0.2546
V_cholerae_MiSeq	4033464	0.005	15	1	5192523.336	0.28736
V_cholerae_MiSeq	4033464	0.005	16	1	5235171.617	0.29793
V_cholerae_MiSeq	4033464	0.005	17	1	5212925.109	0.29242
V_cholerae_MiSeq	4033464	0.005	18	1	5190682.182	0.2869
V_cholerae_MiSeq	4033464	0.005	19	1	5168441.887	0.28139
V_cholerae_MiSeq	4033464	0.005	20	1	5146203.403	0.27588
V_cholerae_MiSeq	4033464	0.005	21	1	5322045.593	0.31947
X_axonopodis_HiSeq	4967469	0.005	11	18	3799196.686	0.23518
X_axonopodis_HiSeq	4967469	0.005	12	4	4236699.106	0.14711
X_axonopodis_HiSeq	4967469	0.005	13	1	4615675.037	0.07082
X_axonopodis_HiSeq	4967469	0.005	14	1	4945083.544	0.0045064
X_axonopodis_HiSeq	4967469	0.005	15	1	5305846.707	0.068119
X_axonopodis_HiSeq	4967469	0.005	16	1	5415824.313	0.090258
X_axonopodis_HiSeq	4967469	0.005	17	1	5465047.601	0.10017
X_axonopodis_HiSeq	4967469	0.005	18	1	5322402.756	0.071452
X_axonopodis_HiSeq	4967469	0.005	19	1	5371833.308	0.081402
X_axonopodis_HiSeq	4967469	0.005	20	1	5446500.958	0.096434
X_axonopodis_HiSeq	4967469	0.005	21	1	5502678.2	0.10774

Table S2. Genome size estimation on real homozygous datesets generated by Pirs using findGSE

DataSet	Ref_length	K	Est.Ref_len	accuracy
A_hydrophila_HiSeq	4673830	16	4896451	0.047631386
A_hydrophila_HiSeq	4673830	21	4918028	0.052247942
A_hydrophila_HiSeq	4673830	20	4918275	0.05230079
A_hydrophila_HiSeq	4673830	19	4919024	0.052461044
A_hydrophila_HiSeq	4673830	18	4921515	0.052994011
A_hydrophila_HiSeq	4673830	17	5005324	0.070925558
A_hydrophila_HiSeq	4673830	15	5019804	0.074023659
B_fragilis_HiSeq	5373121	17	5353337	0.003682031
B_fragilis_HiSeq	5373121	15	5351902	0.003949101
B_fragilis_HiSeq	5373121	20	5350234	0.004259536
B_fragilis_HiSeq	5373121	18	5309280	0.011881549
B_fragilis_HiSeq	5373121	19	5307652	0.012184539
B_fragilis_HiSeq	5373121	21	5306746	0.012353156
B_fragilis_HiSeq	5373121	16	5297822	0.014014015
Human_Chromosome_14	107349540	21	91798688	0.144861841
Human_Chromosome_14	107349540	20	90558888	0.156411029
Human_Chromosome_14	107349540	19	90407702	0.157819381
Human_Chromosome_14	107349540	18	90089976	0.160779115
Human_Chromosome_14	107349540	17	88967839	0.171232229
Human_Chromosome_14	107349540	16	86435606	0.1948209
Human_Chromosome_14	107349540	15	80917106	0.246227734
M_abscessus_HiSeq	5090491	16	5096979	0.001274533
M_abscessus_HiSeq	5090491	17	5110821	0.003993721
M_abscessus_HiSeq	5090491	20	5118027	0.005409301
M_abscessus_HiSeq	5090491	21	5119631	0.005724399
M_abscessus_HiSeq	5090491	18	5122522	0.00629232
M_abscessus_HiSeq	5090491	19	5132446	0.008241838
M_abscessus_HiSeq	5090491	15	5043301	0.009270226
M_abscessus_MiSeq	5090491	21	6421138	0.261398557
M_abscessus_MiSeq_	5090491	20	6444806	0.26604801
M_abscessus_MiSeq	5090491	15	6454602	0.267972382
M_abscessus_MiSeq	5090491	19	6465623	0.270137399
M_abscessus_MiSeq	5090491	18	6492462	0.275409779
M_abscessus_MiSeq	5090491	16	6504755	0.277824673
M_abscessus_MiSeq	5090491	17	6510322	0.278918281
R_sphaeroides_HiSeq	4628173	19	4881932	0.054829195
R_sphaeroides_HiSeq	4628173	21	4972979	0.074501537
R_sphaeroides_HiSeq	4628173	20	4975014	0.074941235

R_sphaeroides_HiSeq	4628173	16	5006515	0.081747592
R_sphaeroides_HiSeq	4628173	18	5007182	0.08189171
R_sphaeroides_HiSeq	4628173	17	5132019	0.108864988
R_sphaeroides_HiSeq	4628173	15	5200655	0.12369503
R_sphaeroides_MiSeq	4628173	21	5426423	0.172476267
R_sphaeroides_MiSeq	4628173	20	5426519	0.172497009
R_sphaeroides_MiSeq	4628173	19	5441724	0.175782323
R_sphaeroides_MiSeq	4628173	18	5474848	0.182939359
R_sphaeroides_MiSeq	4628173	17	5531647	0.195211804
R_sphaeroides_MiSeq	4628173	16	5639663	0.218550603
R_sphaeroides_MiSeq	4628173	15	5720815	0.236084952
Rhodobacter_sphaeroides	4628173	21	5365945	0.159408907
Rhodobacter_sphaeroides	4628173	20	5429441	0.17312836
Rhodobacter_sphaeroides	4628173	19	5466092	0.181047467
Rhodobacter_sphaeroides	4628173	18	6102733	0.318605203
Rhodobacter_sphaeroides	4628173	17	6159575	0.33088694
Rhodobacter_sphaeroides	4628173	16	6206551	0.341036949
Rhodobacter_sphaeroides	4628173	15	6250572	0.350548478
S_aureus_HiSeq	2872915	20	2889550	0.005790286
S_aureus_HiSeq	2872915	18	2893700	0.007234812
S_aureus_HiSeq	2872915	21	2895530	0.007871796
S_aureus_HiSeq	2872915	19	2895768	0.007954638
S_aureus_HiSeq	2872915	17	2899098	0.00911374
S_aureus_HiSeq	2872915	16	2900370	0.009556496
S_aureus_HiSeq	2872915	15	2715947	0.054637189
Staphylococcus_aureus	2903107	16	3614262	0.244963413
Staphylococcus_aureus	2903107	17	3623645	0.248195468
Staphylococcus_aureus	2903107	18	3631107	0.250765817
Staphylococcus_aureus	2903107	19	3647144	0.256289899
Staphylococcus_aureus	2903107	20	3657552	0.259875024
Staphylococcus_aureus	2903107	21	3674528	0.265722552
Staphylococcus_aureus	2903107	15	4262404	0.468221461
V_cholerae_HiSeq	4033464	19	4025526	0.001968035
V_cholerae_HiSeq	4033464	20	4022808	0.002641898
V_cholerae_HiSeq	4033464	17	4044295	0.002685285
V_cholerae_HiSeq	4033464	15	4046202	0.00315808
V_cholerae_HiSeq	4033464	16	4014661	0.00466175
V_cholerae_HiSeq	4033464	21	4011538	0.005436022
V_cholerae_HiSeq	4033464	18	4081915	0.012012255
V_cholerae_MiSeq	4033464	18	4996532	0.238769455
V_cholerae_MiSeq	4033464	20	5006737	0.241299538
V_cholerae_MiSeq	4033464	19	5017333	0.24392656
V_cholerae_MiSeq	4033464	16	5076855	0.258683603
V_cholerae_MiSeq	4033464	17	5098072	0.263943846
V_cholerae_MiSeq	4033464	21	5102700	0.265091247
V_cholerae_MiSeq	4033464	15	5138546	0.273978397
X_axonopodis_HiSeq	4967469	15	5052428	0.017103076
X_axonopodis_HiSeq	4967469	16	5206691	0.048157724
X_axonopodis_HiSeq	4967469	20	5253653	0.057611633
X_axonopodis_HiSeq	4967469	17	5286233	0.064170305
X_axonopodis_HiSeq	4967469	18	5290612	0.06505184
X_axonopodis_HiSeq	4967469	19	5337833	0.074557889
X_axonopodis_HiSeq	4967469	21	5453413	0.097825271

Table S3. Genome size estimation on real homozygous datesets generated by Pirs using GenomeScope

DataSet	Ref_length	K	Est.Ref_len	accuracy
A_hydrophila_HiSeq	4673830	21	4904050	0.049257247
A_hydrophila_HiSeq	4673830	20	4905844	0.049641087
A_hydrophila_HiSeq	4673830	19	4908461	0.050201013
A_hydrophila_HiSeq	4673830	18	4911316	0.050811861
A_hydrophila_HiSeq	4673830	17	4914907	0.051580182
A_hydrophila_HiSeq	4673830	16	4920509	0.05277877
A_hydrophila_HiSeq	4673830	15	4929997	0.054808797
A_hydrophila_HiSeq	4673830	14	4952555	0.059635246
A_hydrophila_HiSeq	4673830	13	4980894	0.065698581
A_hydrophila_HiSeq	4673830	12	2479739	0.469441764
A_hydrophila_HiSeq	4673830	11	53502363	10.44722059

B_fragilis_HiSeq	5373121	14	5271475	0.018917497
B_fragilis_HiSeq	5373121	15	5268430	0.019484207
B_fragilis_HiSeq	5373121	16	5266176	0.019903702
B_fragilis_HiSeq	5373121	17	5264426	0.020229397
B_fragilis_HiSeq	5373121	18	5263064	0.020482881
B_fragilis_HiSeq	5373121	19	5261638	0.020748276
B_fragilis_HiSeq	5373121	20	5260391	0.020980358
B_fragilis_HiSeq	5373121	21	5259519	0.021142647
B_fragilis_HiSeq	5373121	13	2593624	0.517296558
B_fragilis_HiSeq	5373121	11	23392842	3.353678616
Human_Chromosome_14	107349540	17	88076272	0.1795375
Human_Chromosome_14	107349540	18	88067690	0.179617444
Human_Chromosome_14	107349540	19	87951965	0.180695465
Human_Chromosome_14	107349540	16	87854786	0.181600722
Human_Chromosome_14	107349540	20	87803465	0.182078796
Human_Chromosome_14	107349540	21	87662478	0.183392141
Human_Chromosome_14	107349540	15	86913229	0.190371668
Human_Chromosome_14	107349540	14	42107269	0.607755478
Human_Chromosome_14	107349540	13	37951987	0.646463441
M_abscessus_HiSeq	5090491	21	5100382	0.001943035
M_abscessus_HiSeq	5090491	20	5103117	0.002480311
M_abscessus_HiSeq	5090491	19	5106298	0.003105201
M_abscessus_HiSeq	5090491	18	5110165	0.003864853
M_abscessus_HiSeq	5090491	17	5115361	0.00488558
M_abscessus_HiSeq	5090491	16	5123927	0.006568325
M_abscessus_HiSeq	5090491	15	5141341	0.009989213
M_abscessus_HiSeq	5090491	14	5175908	0.016779717
M_abscessus_HiSeq	5090491	13	5221496	0.025735239
M_abscessus_HiSeq	5090491	12	2609577	0.487362417
M_abscessus_HiSeq	5090491	11	2511865	0.506557422
M_abscessus_MiSeq	5090491	21	6294412	0.236503905
M_abscessus_MiSeq	5090491	20	6323993	0.242314936
M_abscessus_MiSeq	5090491	19	6354234	0.24825562
M_abscessus_MiSeq	5090491	18	6385832	0.25446288
M_abscessus_MiSeq	5090491	17	6418710	0.260921589
M_abscessus_MiSeq	5090491	16	6454121	0.267877892
M_abscessus_MiSeq	5090491	15	6495076	0.275923285
M_abscessus_MiSeq	5090491	14	6544912	0.285713303
M_abscessus_MiSeq	5090491	13	6568429	0.290333094
M_abscessus_MiSeq	5090491	12	3255899	0.360395883
R_sphaeroides_HiSeq	4628173	21	4715801	0.018933605
R_sphaeroides_HiSeq	4628173	20	4729106	0.02180839
R_sphaeroides_HiSeq	4628173	19	4746574	0.025582665
R_sphaeroides_HiSeq	4628173	18	4771089	0.030879572
R_sphaeroides_HiSeq	4628173	17	4809333	0.039142876
R_sphaeroides_HiSeq	4628173	16	4850458	0.048028671
R_sphaeroides_HiSeq	4628173	15	4925504	0.064243709
R_sphaeroides_HiSeq	4628173	12	31244968	5.75103718
R_sphaeroides_MiSeq	4628173	21	5104126	0.102838204
R_sphaeroides_MiSeq	4628173	20	5118947	0.106040548
R_sphaeroides_MiSeq	4628173	19	5139913	0.110570629
R_sphaeroides_MiSeq	4628173	18	5173135	0.11774884
R_sphaeroides_MiSeq	4628173	17	5235553	0.131235371
R_sphaeroides_MiSeq	4628173	16	5356005	0.157261191
R_sphaeroides_MiSeq	4628173	15	5587332	0.207243549
R_sphaeroides_MiSeq	4628173	14	5935360	0.282441257
R_sphaeroides_MiSeq	4628173	13	3172538	0.314516117
R_sphaeroides_MiSeq	4628173	12	6460953	0.396005076
Rhodobacter_sphaeroides	4628173	21	4848359	0.047575145
Rhodobacter_sphaeroides	4628173	20	4880189	0.054452589
Rhodobacter_sphaeroides	4628173	19	4933520	0.06597571
Rhodobacter_sphaeroides	4628173	18	5000803	0.080513412
Rhodobacter_sphaeroides	4628173	17	5080612	0.097757582
Rhodobacter_sphaeroides	4628173	16	5182518	0.119776205
Rhodobacter_sphaeroides	4628173	15	5298749	0.144890003
Rhodobacter_sphaeroides	4628173	14	5357460	0.15757557
Rhodobacter_sphaeroides	4628173	13	2608129	0.43646683
Rhodobacter_sphaeroides	4628173	12	2492873	0.461369962

Rhodobacter_sphaeroides	4628173	11	2237922	0.51645671
S_aureus_HiSeq	2872915	15	2610921	0.091194484
S_aureus_HiSeq	2872915	14	2610717	0.091265492
S_aureus_HiSeq	2872915	16	2610347	0.091394281
S_aureus_HiSeq	2872915	17	2609669	0.091630278
S_aureus_HiSeq	2872915	18	2608967	0.091874629
S_aureus_HiSeq	2872915	13	2608310	0.092103317
S_aureus_HiSeq	2872915	19	2608219	0.092134992
S_aureus_HiSeq	2872915	20	2607599	0.0923508
S_aureus_HiSeq	2872915	21	2607090	0.092527972
S_aureus_HiSeq	2872915	12	1280478	0.554293113
S_aureus_HiSeq	2872915	11	54028682	17.80622364
Staphylococcus_aureus_	2903107	13	2124379	0.26823951
Staphylococcus_aureus_	2903107	14	2086632	0.281241787
Staphylococcus_aureus_	2903107	21	2082835	0.282549696
Staphylococcus_aureus_	2903107	20	2078799	0.283939931
Staphylococcus_aureus_	2903107	19	2075073	0.285223383
Staphylococcus_aureus_	2903107	18	2071734	0.28637353
Staphylococcus_aureus_	2903107	15	2069858	0.287019734
Staphylococcus_aureus_	2903107	17	2068895	0.287351448
Staphylococcus_aureus_	2903107	16	2068666	0.287430329
V_cholerae_HiSeq	4033464	13	3826668	0.051270075
V_cholerae_HiSeq	4033464	14	3818117	0.053390089
V_cholerae_HiSeq	4033464	15	3810964	0.055163502
V_cholerae_HiSeq	4033464	16	3806769	0.056203551
V_cholerae_HiSeq	4033464	17	3803823	0.056933941
V_cholerae_HiSeq	4033464	18	3801390	0.057537144
V_cholerae_HiSeq	4033464	19	3799256	0.058066218
V_cholerae_HiSeq	4033464	20	3797154	0.058587358
V_cholerae_HiSeq	4033464	21	3795060	0.059106515
V_cholerae_HiSeq	4033464	12	1900652	0.528779233
V_cholerae_HiSeq	4033464	11	1815368	0.549923341
V_cholerae_MiSeq	4033464	21	4663517	0.156206427
V_cholerae_MiSeq	4033464	20	4681966	0.160780411
V_cholerae_MiSeq	4033464	19	4700100	0.165276298
V_cholerae_MiSeq	4033464	18	4718196	0.169762765
V_cholerae_MiSeq	4033464	17	4736516	0.174304766
V_cholerae_MiSeq	4033464	16	4755166	0.178928583
V_cholerae_MiSeq	4033464	15	4774339	0.183682066
V_cholerae_MiSeq	4033464	14	4793716	0.188486125
V_cholerae_MiSeq	4033464	13	4796915	0.18927924
V_cholerae_MiSeq	4033464	12	2379531	0.410052749
V_cholerae_MiSeq	4033464	11	2206942	0.452842024
X_axonopodis_HiSeq	4967469	13	5326135	0.072202967
X_axonopodis_HiSeq	4967469	14	5359820	0.078984086
X_axonopodis_HiSeq	4967469	15	5368159	0.080662808
X_axonopodis_HiSeq	4967469	21	5368658	0.080763262
X_axonopodis_HiSeq	4967469	20	5369728	0.080978663
X_axonopodis_HiSeq	4967469	19	5370851	0.081204734
X_axonopodis_HiSeq	4967469	16	5371657	0.08136699
X_axonopodis_HiSeq	4967469	18	5371945	0.081424967
X_axonopodis_HiSeq	4967469	17	5372467	0.081530051
X_axonopodis_HiSeq	4967469	12	2606991	0.475187263
X_axonopodis_HiSeq	4967469	11	40456185	7.144224956

Table S4. Genome size estimation on simulated heterozygous datesets generated by Pirs using GEST

DataSet	Ref_Length	Error_rate	SNP_Rate	K	L	Est.RefLen	Accurancy
test2020000.300.75-0.02-0.05	2020000	0.02	0.05	11	279	2096833.895	0.038037
test2020000.300.75-0.02-0.05	2020000	0.02	0.05	12	59	11046204.45	4.4684
test2020000.300.75-0.02-0.05	2020000	0.02	0.05	14	2	41296162.27	19.4436
test2020000.300.75-0.02-0.05	2020000	0.02	0.05	15	1	45866630.28	21.7063
test2020000.300.75-0.02-0.05	2020000	0.02	0.05	16	1	48450397.55	22.9853
test2020000.300.75-0.02-0.05	2020000	0.02	0.05	17	1	50309401.67	23.9056
test2020000.300.75-0.02-0.05	2020000	0.02	0.05	18	1	51541402.77	24.5155
test2020000.300.75-0.02-0.05	2020000	0.02	0.05	19	1	52861260.94	25.1689
test2020000.300.75-0.02-0.05	2020000	0.02	0.05	21	1	53599519.01	25.5344
test2020000.300.75-0.02-0.05	2020000	0.02	0.05	20	1	54277993.94	25.8703

test2020000.300.75-0.02-0.05	2020000	0.02	0.05	13	7	174989347.9	85.6284
test2020000.350.75-0.04-0.045	2020000	0.04	0.045	11	19	3319895.779	0.64351
test2020000.350.75-0.04-0.045	2020000	0.04	0.045	12	4	3737025.966	0.85001
test2020000.350.75-0.04-0.045	2020000	0.04	0.045	13	1	4726725.444	1.34
test2020000.350.75-0.04-0.045	2020000	0.04	0.045	14	1	5432134.334	1.6892
test2020000.350.75-0.04-0.045	2020000	0.04	0.045	16	1	5659222.642	1.8016
test2020000.350.75-0.04-0.045	2020000	0.04	0.045	15	1	5726594.34	1.8349
test2020000.350.75-0.04-0.045	2020000	0.04	0.045	17	1	5826371.56	1.8843
test2020000.350.75-0.04-0.045	2020000	0.04	0.045	18	1	6176703.109	2.0578
test2020000.350.75-0.04-0.045	2020000	0.04	0.045	20	1	6314131.805	2.1258
test2020000.350.75-0.04-0.045	2020000	0.04	0.045	19	1	6393058.453	2.1649
test2020000.350.75-0.04-0.045	2020000	0.04	0.045	21	1	6545200.319	2.2402
test2020000.400.55-0.05-0.02	2020000	0.05	0.02	11	253	2254229.896	0.11596
test2020000.400.55-0.05-0.02	2020000	0.05	0.02	12	56	11757138.36	4.8204
test2020000.400.55-0.05-0.02	2020000	0.05	0.02	13	7	23772144.79	10.7684
test2020000.400.55-0.05-0.02	2020000	0.05	0.02	14	2	30228781.47	13.9647
test2020000.400.55-0.05-0.02	2020000	0.05	0.02	15	1	33319831.53	15.495
test2020000.400.55-0.05-0.02	2020000	0.05	0.02	16	1	35666253.74	16.6566
test2020000.400.55-0.05-0.02	2020000	0.05	0.02	18	1	36588538.5	17.1131
test2020000.400.55-0.05-0.02	2020000	0.05	0.02	17	1	37034740.19	17.334
test2020000.400.55-0.05-0.02	2020000	0.05	0.02	19	1	37478961.69	17.5539
test2020000.400.55-0.05-0.02	2020000	0.05	0.02	20	1	38432854.04	18.0262
test2020000.400.55-0.05-0.02	2020000	0.05	0.02	21	1	39456725.68	18.533
test2040000.250.50-0.01-0.02	2040000	0.01	0.02	11	196	2167544.592	0.062522
test2040000.250.50-0.01-0.02	2040000	0.01	0.02	12	31	12623917.52	5.1882
test2040000.250.50-0.01-0.02	2040000	0.01	0.02	14	1	32817640.85	15.0871
test2040000.250.50-0.01-0.02	2040000	0.01	0.02	15	1	36173415.24	16.7321
test2040000.250.50-0.01-0.02	2040000	0.01	0.02	16	1	38720790.21	17.9808
test2040000.250.50-0.01-0.02	2040000	0.01	0.02	17	1	40206476.85	18.7091
test2040000.250.50-0.01-0.02	2040000	0.01	0.02	18	1	41191072.62	19.1917
test2040000.250.50-0.01-0.02	2040000	0.01	0.02	20	1	41724328.25	19.4531
test2040000.250.50-0.01-0.02	2040000	0.01	0.02	19	1	42245882.36	19.7088
test2040000.250.50-0.01-0.02	2040000	0.01	0.02	21	1	42835886.51	19.998
test2040000.250.50-0.01-0.02	2040000	0.01	0.02	13	5	184260131	89.3236
test2040000.250.85-0.03-0.005	2040000	0.03	0.005	11	274	2181490.233	0.069358
test2040000.250.85-0.03-0.005	2040000	0.03	0.005	12	59	10253328.27	4.0261
test2040000.250.85-0.03-0.005	2040000	0.03	0.005	13	7	27129523.2	12.2988
test2040000.250.85-0.03-0.005	2040000	0.03	0.005	14	2	36496347.45	16.8904
test2040000.250.85-0.03-0.005	2040000	0.03	0.005	15	1	41024513.08	19.1101
test2040000.250.85-0.03-0.005	2040000	0.03	0.005	16	1	43913508.14	20.5262
test2040000.250.85-0.03-0.005	2040000	0.03	0.005	17	1	45598435.33	21.3522
test2040000.250.85-0.03-0.005	2040000	0.03	0.005	18	1	46715072	21.8995
test2040000.250.85-0.03-0.005	2040000	0.03	0.005	20	1	47319840.8	22.196
test2040000.250.85-0.03-0.005	2040000	0.03	0.005	19	1	47911338.81	22.486
test2040000.250.85-0.03-0.005	2040000	0.03	0.005	21	1	48580466.48	22.814
test2040000.450.95-0.02-0.045	2040000	0.02	0.045	11	114	2389204.49	0.17118
test2040000.450.95-0.02-0.045	2040000	0.02	0.045	12	14	14237070.14	5.979
test2040000.450.95-0.02-0.045	2040000	0.02	0.045	13	3	15912739.81	6.8004
test2040000.450.95-0.02-0.045	2040000	0.02	0.045	14	1	18731199.76	8.182
test2040000.450.95-0.02-0.045	2040000	0.02	0.045	15	1	19726126.49	8.6697
test2040000.450.95-0.02-0.045	2040000	0.02	0.045	17	1	20863891.62	9.2274
test2040000.450.95-0.02-0.045	2040000	0.02	0.045	16	1	21115263.81	9.3506
test2040000.450.95-0.02-0.045	2040000	0.02	0.045	18	1	21326623.57	9.4542
test2040000.450.95-0.02-0.045	2040000	0.02	0.045	20	1	21914498.46	9.7424
test2040000.450.95-0.02-0.045	2040000	0.02	0.045	19	1	22188429.69	9.8767
test2040000.450.95-0.02-0.045	2040000	0.02	0.045	21	1	22468742.02	10.0141
test2060000.450.90-0.03-0.025	2060000	0.03	0.025	11	84	2641333.975	0.2822
test2060000.450.90-0.03-0.025	2060000	0.03	0.025	12	11	12274502.07	4.9585
test2060000.450.90-0.03-0.025	2060000	0.03	0.025	13	2	13557180	5.5812
test2060000.450.90-0.03-0.025	2060000	0.03	0.025	14	1	15255132.15	6.4054
test2060000.450.90-0.03-0.025	2060000	0.03	0.025	15	1	16857384.96	7.1832
test2060000.450.90-0.03-0.025	2060000	0.03	0.025	16	1	17012931.76	7.2587
test2060000.450.90-0.03-0.025	2060000	0.03	0.025	17	1	17450084.53	7.4709
test2060000.450.90-0.03-0.025	2060000	0.03	0.025	18	1	17917328	7.6977
test2060000.450.90-0.03-0.025	2060000	0.03	0.025	20	1	18619303	8.0385
test2060000.450.90-0.03-0.025	2060000	0.03	0.025	19	1	18852044.29	8.1515
test2060000.450.90-0.03-0.025	2060000	0.03	0.025	21	1	19200308.71	8.3205
test2120000.300.80-0.01-0.045	2120000	0.01	0.045	11	123	2273719.944	0.072509

test2120000.300.80-0.01-0.045	2120000	0.01	0.045	12	14	16894405.44	6.9691
test2120000.300.80-0.01-0.045	2120000	0.01	0.045	14	1	22200769.5	9.4721
test2120000.300.80-0.01-0.045	2120000	0.01	0.045	15	1	23740903.06	10.1985
test2120000.300.80-0.01-0.045	2120000	0.01	0.045	16	1	24541491.72	10.5762
test2120000.300.80-0.01-0.045	2120000	0.01	0.045	18	1	24738952	10.6693
test2120000.300.80-0.01-0.045	2120000	0.01	0.045	17	1	25040646.54	10.8116
test2120000.300.80-0.01-0.045	2120000	0.01	0.045	19	1	25627173.18	11.0883
test2120000.300.80-0.01-0.045	2120000	0.01	0.045	21	1	25888428.47	11.2115
test2120000.300.80-0.01-0.045	2120000	0.01	0.045	20	1	26216130.1	11.3661
test2120000.300.80-0.01-0.045	2120000	0.01	0.045	13	2	230602374	107.7747
test2160000.300.95-0.05-0.045	2160000	0.05	0.045	11	229	2278641.885	0.054927
test2160000.300.95-0.05-0.045	2160000	0.05	0.045	12	50	11421021.12	4.2875
test2160000.300.95-0.05-0.045	2160000	0.05	0.045	13	6	21938609.56	9.1568
test2160000.300.95-0.05-0.045	2160000	0.05	0.045	14	2	27563961.92	11.7611
test2160000.300.95-0.05-0.045	2160000	0.05	0.045	15	1	30614613.58	13.1734
test2160000.300.95-0.05-0.045	2160000	0.05	0.045	16	1	31798829.24	13.7217
test2160000.300.95-0.05-0.045	2160000	0.05	0.045	18	1	32662910.74	14.1217
test2160000.300.95-0.05-0.045	2160000	0.05	0.045	17	1	33061238.92	14.3061
test2160000.300.95-0.05-0.045	2160000	0.05	0.045	19	1	33417291.38	14.471
test2160000.300.95-0.05-0.045	2160000	0.05	0.045	21	1	34412128.69	14.9315
test2160000.300.95-0.05-0.045	2160000	0.05	0.045	20	1	34847725.26	15.1332
test2180000.300.80-0.01-0.015	2180000	0.01	0.015	11	26	3363592.714	0.54293
test2180000.300.80-0.01-0.015	2180000	0.01	0.015	13	1	9597744.37	3.4026
test2180000.300.80-0.01-0.015	2180000	0.01	0.015	14	1	10980455.06	4.0369
test2180000.300.80-0.01-0.015	2180000	0.01	0.015	15	1	11549498.91	4.2979
test2180000.300.80-0.01-0.015	2180000	0.01	0.015	17	1	12028950.92	4.5179
test2180000.300.80-0.01-0.015	2180000	0.01	0.015	16	1	12173878.04	4.5843
test2180000.300.80-0.01-0.015	2180000	0.01	0.015	18	1	12702922.92	4.827
test2180000.300.80-0.01-0.015	2180000	0.01	0.015	19	1	13125022.6	5.0207
test2180000.300.80-0.01-0.015	2180000	0.01	0.015	21	1	13412907.03	5.1527
test2180000.300.80-0.01-0.015	2180000	0.01	0.015	20	1	13582690.66	5.2306
test2180000.300.80-0.01-0.015	2180000	0.01	0.015	12	4	55709347.69	24.5547

Table S5. Genome size estimation on simulated heterozygous datesets generated by Pirs using findGSE

DataSet	Ref_length	Insert_Size	Depth	Error_rate	SNP_Rate	K	Est.Ref_Len	Accuracy
test2020000.300.75-0.02-0.05	2020000	300	75	0.02	0.05	16	17950765	7.886517327
test2020000.300.75-0.02-0.05	2020000	300	75	0.02	0.05	15	19465077	8.636176733
test2020000.300.75-0.02-0.05	2020000	300	75	0.02	0.05	17	19561857	8.684087624
test2020000.300.75-0.02-0.05	2020000	300	75	0.02	0.05	21	19788186	8.796131683
test2020000.300.75-0.02-0.05	2020000	300	75	0.02	0.05	18	19801941	8.802941089
test2020000.300.75-0.02-0.05	2020000	300	75	0.02	0.05	19	20076627	8.938924257
test2020000.300.75-0.02-0.05	2020000	300	75	0.02	0.05	20	20397504	9.097774257
test2020000.350.75-0.04-0.045	2020000	350	75	0.04	0.045	16	2001913	0.00895396
test2020000.350.75-0.04-0.045	2020000	350	75	0.04	0.045	17	2057557	0.018592574
test2020000.350.75-0.04-0.045	2020000	350	75	0.04	0.045	18	2092187	0.035736139
test2020000.350.75-0.04-0.045	2020000	350	75	0.04	0.045	15	2102599	0.040890594
test2020000.350.75-0.04-0.045	2020000	350	75	0.04	0.045	19	2132821	0.05585198
test2020000.400.55-0.05-0.02	2020000	400	55	0.05	0.02	15	13606810	5.736044554
test2020000.400.55-0.05-0.02	2020000	400	55	0.05	0.02	18	13776236	5.819918812
test2020000.400.55-0.05-0.02	2020000	400	55	0.05	0.02	19	13930184	5.896130693
test2020000.400.55-0.05-0.02	2020000	400	55	0.05	0.02	20	14114218	5.987236634
test2020000.400.55-0.05-0.02	2020000	400	55	0.05	0.02	21	14179165	6.019388614
test2020000.400.55-0.05-0.02	2020000	400	55	0.05	0.02	17	14199705	6.029556931
test2020000.400.55-0.05-0.02	2020000	400	55	0.05	0.02	16	14260709	6.059756931
test2040000.250.50-0.01-0.02	2040000	250	50	0.01	0.02	20	14652436	6.182566667
test2040000.250.50-0.01-0.02	2040000	250	50	0.01	0.02	15	14815924	6.262707843
test2040000.250.50-0.01-0.02	2040000	250	50	0.01	0.02	16	15371836	6.535213725
test2040000.250.50-0.01-0.02	2040000	250	50	0.01	0.02	17	15519528	6.607611765
test2040000.250.50-0.01-0.02	2040000	250	50	0.01	0.02	18	15678703	6.685638725
test2040000.250.50-0.01-0.02	2040000	250	50	0.01	0.02	21	15726203	6.708923039
test2040000.250.50-0.01-0.02	2040000	250	50	0.01	0.02	19	15900415	6.794321078
test2040000.250.85-0.03-0.005	2040000	250	85	0.03	0.005	15	16710051	7.191201471
test2040000.250.85-0.03-0.005	2040000	250	85	0.03	0.005	20	17378200	7.51872549
test2040000.250.85-0.03-0.005	2040000	250	85	0.03	0.005	16	17407081	7.532882843
test2040000.250.85-0.03-0.005	2040000	250	85	0.03	0.005	17	17533148	7.594680392
test2040000.250.85-0.03-0.005	2040000	250	85	0.03	0.005	21	17600131	7.627515196

test2040000.250.85-0.03-0.005	2040000	250	85	0.03	0.005	18	17745034	7.698546078
test2040000.250.85-0.03-0.005	2040000	250	85	0.03	0.005	19	18017012	7.831868627
test2040000.450.95-0.02-0.045	2040000	450	95	0.02	0.045	15	8168812	3.004319608
test2040000.450.95-0.02-0.045	2040000	450	95	0.02	0.045	20	8249081	3.043667157
test2040000.450.95-0.02-0.045	2040000	450	95	0.02	0.045	17	8254896	3.046517647
test2040000.450.95-0.02-0.045	2040000	450	95	0.02	0.045	18	8362929	3.099475
test2040000.450.95-0.02-0.045	2040000	450	95	0.02	0.045	21	8408219	3.12167598
test2040000.450.95-0.02-0.045	2040000	450	95	0.02	0.045	16	8580791	3.206270098
test2040000.450.95-0.02-0.045	2040000	450	95	0.02	0.045	19	8680004	3.254903922
test2060000.450.90-0.03-0.025	2060000	450	90	0.03	0.025	15	6819239	2.310310194
test2060000.450.90-0.03-0.025	2060000	450	90	0.03	0.025	16	6835583	2.318244175
test2060000.450.90-0.03-0.025	2060000	450	90	0.03	0.025	17	7037805	2.416410194
test2060000.450.90-0.03-0.025	2060000	450	90	0.03	0.025	20	7100026	2.446614563
test2060000.450.90-0.03-0.025	2060000	450	90	0.03	0.025	18	7144367	2.46813932
test2060000.450.90-0.03-0.025	2060000	450	90	0.03	0.025	21	7258986	2.523779612
test2060000.450.90-0.03-0.025	2060000	450	90	0.03	0.025	19	7294928	2.541227184
test2120000.300.80-0.01-0.045	2120000	300	80	0.01	0.045	21	9938322	3.687887736
test2120000.300.80-0.01-0.045	2120000	300	80	0.01	0.045	18	9950779	3.693763679
test2120000.300.80-0.01-0.045	2120000	300	80	0.01	0.045	19	10089367	3.759135377
test2120000.300.80-0.01-0.045	2120000	300	80	0.01	0.045	15	10096838	3.762659434
test2120000.300.80-0.01-0.045	2120000	300	80	0.01	0.045	16	10125798	3.776319811
test2120000.300.80-0.01-0.045	2120000	300	80	0.01	0.045	17	10235901	3.828255189
test2120000.300.80-0.01-0.045	2120000	300	80	0.01	0.045	20	10239778	3.830083962
test2160000.300.95-0.05-0.045	2160000	300	95	0.05	0.045	18	12721065	4.889381944
test2160000.300.95-0.05-0.045	2160000	300	95	0.05	0.045	21	12723218	4.890378704
test2160000.300.95-0.05-0.045	2160000	300	95	0.05	0.045	19	12896124	4.970427778
test2160000.300.95-0.05-0.045	2160000	300	95	0.05	0.045	15	12948645	4.994743056
test2160000.300.95-0.05-0.045	2160000	300	95	0.05	0.045	16	12978020	5.008342593
test2160000.300.95-0.05-0.045	2160000	300	95	0.05	0.045	20	13092015	5.061118056
test2160000.300.95-0.05-0.045	2160000	300	95	0.05	0.045	17	13102666	5.066049074
test2180000.300.80-0.01-0.015	2180000	300	80	0.01	0.015	17	4990686	1.289305505
test2180000.300.80-0.01-0.015	2180000	300	80	0.01	0.015	15	5028957	1.306861009
test2180000.300.80-0.01-0.015	2180000	300	80	0.01	0.015	18	5109524	1.343818349
test2180000.300.80-0.01-0.015	2180000	300	80	0.01	0.015	16	5123500	1.350229358
test2180000.300.80-0.01-0.015	2180000	300	80	0.01	0.015	19	5247497	1.407108716
test2180000.300.80-0.01-0.015	2180000	300	80	0.01	0.015	21	5308828	1.435242202
test2180000.300.80-0.01-0.015	2180000	300	80	0.01	0.015	20	5400593	1.477336239

Table S6. Genome size estimation on simulated heterozygous datesets generated by Pirs using GenomeScope

DataSet	Ref_length	Insert_Size	Depth	Error_rate	SNP_Rate	K	Est.Ref_Len	Accuracy
test2020000.300.75-0.02-0.05	2020000	300	75	0.02	0.05	11	2076255	0.02784901
test2020000.300.75-0.02-0.05	2020000	300	75	0.02	0.05	12	7636451	2.780421287
test2020000.300.75-0.02-0.05	2020000	300	75	0.02	0.05	13	17010442	7.421010891
test2020000.300.75-0.02-0.05	2020000	300	75	0.02	0.05	21	18386476	8.102215842
test2020000.300.75-0.02-0.05	2020000	300	75	0.02	0.05	15	18425001	8.121287624
test2020000.300.75-0.02-0.05	2020000	300	75	0.02	0.05	20	18457492	8.137372277
test2020000.300.75-0.02-0.05	2020000	300	75	0.02	0.05	14	18458904	8.138071287
test2020000.300.75-0.02-0.05	2020000	300	75	0.02	0.05	16	18485867	8.151419307
test2020000.300.75-0.02-0.05	2020000	300	75	0.02	0.05	19	18506226	8.16149802
test2020000.300.75-0.02-0.05	2020000	300	75	0.02	0.05	18	18540209	8.178321287
test2020000.300.75-0.02-0.05	2020000	300	75	0.02	0.05	17	18561048	8.188637624
test2020000.350.75-0.04-0.045	2020000	350	75	0.04	0.045	20	2016934	0.001517822
test2020000.350.75-0.04-0.045	2020000	350	75	0.04	0.045	19	2015851	0.00205396
test2020000.350.75-0.04-0.045	2020000	350	75	0.04	0.045	18	2015225	0.002363861
test2020000.350.75-0.04-0.045	2020000	350	75	0.04	0.045	16	2014683	0.002632178
test2020000.350.75-0.04-0.045	2020000	350	75	0.04	0.045	15	2012620	0.003653465
test2020000.350.75-0.04-0.045	2020000	350	75	0.04	0.045	14	2009099	0.005396535
test2020000.350.75-0.04-0.045	2020000	350	75	0.04	0.045	13	1996468	0.011649505
test2020000.350.75-0.04-0.045	2020000	350	75	0.04	0.045	12	1936578	0.04129802
test2020000.350.75-0.04-0.045	2020000	350	75	0.04	0.045	17	4028963	0.994536139
test2020000.350.75-0.04-0.045	2020000	350	75	0.04	0.045	21	4033882	0.996971287
test2020000.400.55-0.05-0.02	2020000	400	55	0.05	0.02	11	2063510	0.021539604
test2020000.400.55-0.05-0.02	2020000	400	55	0.05	0.02	13	12653464	5.264091089
test2020000.400.55-0.05-0.02	2020000	400	55	0.05	0.02	20	12822819	5.347930198
test2020000.400.55-0.05-0.02	2020000	400	55	0.05	0.02	19	12923154	5.39760099

test2020000.400.55-0.05-0.02	2020000	400	55	0.05	0.02	18	13005336	5.438285149
test2020000.400.55-0.05-0.02	2020000	400	55	0.05	0.02	15	13036634	5.453779208
test2020000.400.55-0.05-0.02	2020000	400	55	0.05	0.02	16	13038222	5.454565347
test2020000.400.55-0.05-0.02	2020000	400	55	0.05	0.02	14	13049433	5.460115347
test2020000.400.55-0.05-0.02	2020000	400	55	0.05	0.02	17	13070537	5.470562871
test2020000.400.55-0.05-0.02	2020000	400	55	0.05	0.02	21	13489228	5.677835644
test2040000.250.50-0.01-0.02	2040000	250	50	0.01	0.02	11	2067485	0.013473039
test2040000.250.50-0.01-0.02	2040000	250	50	0.01	0.02	13	13638108	5.685347059
test2040000.250.50-0.01-0.02	2040000	250	50	0.01	0.02	21	14092410	5.908044118
test2040000.250.50-0.01-0.02	2040000	250	50	0.01	0.02	14	14182567	5.952238725
test2040000.250.50-0.01-0.02	2040000	250	50	0.01	0.02	20	14196125	5.958884804
test2040000.250.50-0.01-0.02	2040000	250	50	0.01	0.02	15	14206364	5.963903922
test2040000.250.50-0.01-0.02	2040000	250	50	0.01	0.02	16	14252808	5.986670588
test2040000.250.50-0.01-0.02	2040000	250	50	0.01	0.02	19	14267814	5.994026471
test2040000.250.50-0.01-0.02	2040000	250	50	0.01	0.02	18	14319090	6.019161765
test2040000.250.50-0.01-0.02	2040000	250	50	0.01	0.02	17	14353296	6.035929412
test2040000.250.50-0.01-0.02	2040000	250	50	0.01	0.02	12	15270664	6.485619608
test2040000.250.85-0.03-0.005	2040000	250	85	0.03	0.005	11	2072365	0.015865196
test2040000.250.85-0.03-0.005	2040000	250	85	0.03	0.005	12	7465620	2.659617647
test2040000.250.85-0.03-0.005	2040000	250	85	0.03	0.005	13	15349418	6.52422451
test2040000.250.85-0.03-0.005	2040000	250	85	0.03	0.005	21	16087140	6.885852941
test2040000.250.85-0.03-0.005	2040000	250	85	0.03	0.005	20	16180472	6.931603922
test2040000.250.85-0.03-0.005	2040000	250	85	0.03	0.005	19	16248273	6.964839706
test2040000.250.85-0.03-0.005	2040000	250	85	0.03	0.005	15	16260746	6.970953922
test2040000.250.85-0.03-0.005	2040000	250	85	0.03	0.005	16	16266403	6.973726961
test2040000.250.85-0.03-0.005	2040000	250	85	0.03	0.005	18	16299451	6.989926961
test2040000.250.85-0.03-0.005	2040000	250	85	0.03	0.005	17	16337698	7.00867549
test2040000.250.85-0.03-0.005	2040000	250	85	0.03	0.005	14	16351599	7.015489706
test2040000.450.95-0.02-0.045	2040000	450	95	0.02	0.045	11	1991727	0.023663235
test2040000.450.95-0.02-0.045	2040000	450	95	0.02	0.045	21	7634658	2.742479412
test2040000.450.95-0.02-0.045	2040000	450	95	0.02	0.045	20	7720156	2.784390196
test2040000.450.95-0.02-0.045	2040000	450	95	0.02	0.045	18	7757046	2.802473529
test2040000.450.95-0.02-0.045	2040000	450	95	0.02	0.045	19	7797366	2.822238235
test2040000.450.95-0.02-0.045	2040000	450	95	0.02	0.045	17	7829491	2.837985784
test2040000.450.95-0.02-0.045	2040000	450	95	0.02	0.045	15	7886220	2.865794118
test2040000.450.95-0.02-0.045	2040000	450	95	0.02	0.045	14	7887419	2.866381863
test2040000.450.95-0.02-0.045	2040000	450	95	0.02	0.045	16	7890938	2.868106863
test2040000.450.95-0.02-0.045	2040000	450	95	0.02	0.045	13	7905859	2.875421078
test2060000.450.90-0.03-0.025	2060000	450	90	0.03	0.025	11	1931808	0.062229126
test2060000.450.90-0.03-0.025	2060000	450	90	0.03	0.025	14	5869507	1.849275243
test2060000.450.90-0.03-0.025	2060000	450	90	0.03	0.025	13	5941032	1.883996117
test2060000.450.90-0.03-0.025	2060000	450	90	0.03	0.025	15	6406390	2.109898058
test2060000.450.90-0.03-0.025	2060000	450	90	0.03	0.025	16	6498915	2.154813107
test2060000.450.90-0.03-0.025	2060000	450	90	0.03	0.025	17	6583330	2.195791262
test2060000.450.90-0.03-0.025	2060000	450	90	0.03	0.025	18	6661149	2.233567476
test2060000.450.90-0.03-0.025	2060000	450	90	0.03	0.025	19	6730623	2.267292718
test2060000.450.90-0.03-0.025	2060000	450	90	0.03	0.025	20	6795920	2.298990291
test2060000.450.90-0.03-0.025	2060000	450	90	0.03	0.025	12	46948877	21.79071699
test2120000.300.80-0.01-0.045	2120000	300	80	0.01	0.045	11	2022445	0.046016509
test2120000.300.80-0.01-0.045	2120000	300	80	0.01	0.045	21	9623376	3.539328302
test2120000.300.80-0.01-0.045	2120000	300	80	0.01	0.045	20	9708646	3.57955
test2120000.300.80-0.01-0.045	2120000	300	80	0.01	0.045	18	9779861	3.613141981
test2120000.300.80-0.01-0.045	2120000	300	80	0.01	0.045	19	9786496	3.616271698
test2120000.300.80-0.01-0.045	2120000	300	80	0.01	0.045	17	9854450	3.648325472
test2120000.300.80-0.01-0.045	2120000	300	80	0.01	0.045	13	9873323	3.65722783
test2120000.300.80-0.01-0.045	2120000	300	80	0.01	0.045	16	9922339	3.680348585
test2120000.300.80-0.01-0.045	2120000	300	80	0.01	0.045	15	9939912	3.688637736
test2120000.300.80-0.01-0.045	2120000	300	80	0.01	0.045	14	9954014	3.695289623
test2160000.300.95-0.05-0.045	2160000	300	95	0.05	0.045	11	2051880	0.050055556
test2160000.300.95-0.05-0.045	2160000	300	95	0.05	0.045	13	11796764	4.461464815
test2160000.300.95-0.05-0.045	2160000	300	95	0.05	0.045	21	11977603	4.545186574
test2160000.300.95-0.05-0.045	2160000	300	95	0.05	0.045	19	12048053	4.577802315
test2160000.300.95-0.05-0.045	2160000	300	95	0.05	0.045	20	12087827	4.596216204
test2160000.300.95-0.05-0.045	2160000	300	95	0.05	0.045	18	12135440	4.618259259
test2160000.300.95-0.05-0.045	2160000	300	95	0.05	0.045	16	12187741	4.642472685
test2160000.300.95-0.05-0.045	2160000	300	95	0.05	0.045	15	12198780	4.647583333
test2160000.300.95-0.05-0.045	2160000	300	95	0.05	0.045	17	12204773	4.65035787
test2160000.300.95-0.05-0.045	2160000	300	95	0.05	0.045	14	12238146	4.665808333

test2160000.300.95-0.05-0.045	2160000	300	95	0.05	0.045	12	13077922	5.054593519
test2180000.300.80-0.01-0.015	2180000	300	80	0.01	0.015	11	3691971	0.693564679
test2180000.300.80-0.01-0.015	2180000	300	80	0.01	0.015	12	3955818	0.814595413
test2180000.300.80-0.01-0.015	2180000	300	80	0.01	0.015	13	4319582	0.981459633
test2180000.300.80-0.01-0.015	2180000	300	80	0.01	0.015	14	4527943	1.077038073
test2180000.300.80-0.01-0.015	2180000	300	80	0.01	0.015	15	4669217	1.141842661
test2180000.300.80-0.01-0.015	2180000	300	80	0.01	0.015	17	4840635	1.220474771
test2180000.300.80-0.01-0.015	2180000	300	80	0.01	0.015	16	9214428	3.226801835
test2180000.300.80-0.01-0.015	2180000	300	80	0.01	0.015	18	9514674	3.364529358
test2180000.300.80-0.01-0.015	2180000	300	80	0.01	0.015	19	9658848	3.43066422
test2180000.300.80-0.01-0.015	2180000	300	80	0.01	0.015	20	9799118	3.495008257
test2180000.300.80-0.01-0.015	2180000	300	80	0.01	0.015	21	9929649	3.554884862

Table S7. Genome size estimation on real heterozygous datesets using GEST

DataSet	Ref_Length	Error_rate	K	L	minX	Est.RefLen	Accurancy
s.venezuelensis	52178999	0.05	11	641	6311	411089.7869	0.99212
s.venezuelensis	52178999	0.05	12	193	4058	631731.8792	0.98789
s.venezuelensis	52178999	0.05	13	90	2382	1067350.663	0.97954
s.venezuelensis	52178999	0.05	14	54	22	51745878.23	0.0083007
s.venezuelensis	52178999	0.05	15	41	20	52737548.17	0.010704
s.venezuelensis	52178999	0.05	16	35	19	54072677.44	0.036292
s.venezuelensis	52178999	0.05	17	31	18	54909651.21	0.052332
s.venezuelensis	52178999	0.05	18	28	17	103954513.3	0.99227
s.venezuelensis	52178999	0.05	19	25	17	104647845.3	1.0056
s.venezuelensis	52178999	0.05	20	24	16	107480609.1	1.0598
s.venezuelensis	52178999	0.05	21	22	16	106295379	1.0371
s.venezuelensis	52178999	0.05	22	20	16	107079753.8	1.0522
s.venezuelensis	52178999	0.05	23	19	15	108030283.3	1.0704
s.venezuelensis	52178999	0.05	24	17	15	108916031.7	1.0874
s.venezuelensis	52178999	0.05	25	15	15	107659893.7	1.0633
c.elegans	100286401	0.05	14	53	18	98744368.89	0.015376
c.elegans	100286401	0.05	15	46	16	102234588.4	0.019426
c.elegans	100286401	0.05	16	43	15	101986553.8	0.016953

Table S8. Genome size estimation on real heterozygous datesets using findGSE

DataSet	Ref_Length	Error_rate	K	L	Est.RefLen	Accurancy
s.venezuelensis	52178999	0.05	15	41	53632603	0.027858028
s.venezuelensis	52178999	0.05	16	35	54190400	0.038548095
s.venezuelensis	52178999	0.05	17	31	54801900	0.050267369
s.venezuelensis	52178999	0.05	18	28	59527681	0.14083601
s.venezuelensis	52178999	0.05	19	25	60304831	0.155729933
s.venezuelensis	52178999	0.05	20	24	61134185	0.171624335
s.venezuelensis	52178999	0.05	21	22	60346876	0.156535717
s.venezuelensis	52178999	0.05	22	20	61244626	0.173740914
s.venezuelensis	52178999	0.05	23	19	60463873	0.158777941
s.venezuelensis	52178999	0.05	24	17	61443993	0.177561743
s.venezuelensis	52178999	0.05	25	15	60649855	0.162342248
c.elegans	100286401	0.05	15	46	106793837	0.064888519
c.elegans	100286401	0.05	16	43	107492345	0.07185365

Table S9. Genome size estimation on real heterozygous datesets using GenomeScope

DataSet	Ref_Length	Error_rate	K	Est.RefLen	Accurancy
s.venezuelensis	52178999	0.05	13	50789029	0.026638495
s.venezuelensis	52178999	0.05	14	52291476	0.002155599
s.venezuelensis	52178999	0.05	15	53026908	0.016250005
s.venezuelensis	52178999	0.05	16	53533664	0.025961882
s.venezuelensis	52178999	0.05	17	53948910	0.033919988
s.venezuelensis	52178999	0.05	18	54268933	0.040053164
s.venezuelensis	52178999	0.05	19	54483758	0.044170242
s.venezuelensis	52178999	0.05	20	54599819	0.046394527
s.venezuelensis	52178999	0.05	21	54640585	0.0471758

s.venezuelensis	52178999	0.05	22	54638327	0.047132525
s.venezuelensis	52178999	0.05	23	54614921	0.046683954
s.venezuelensis	52178999	0.05	24	54580209	0.046018706
s.venezuelensis	52178999	0.05	25	54536527	0.045181549
c.elegans	100286401	0.05	14	51992730	0.481557524
c.elegans	100286401	0.05	15	104353383	0.040553674
c.elegans	100286401	0.05	16	104715846	0.044167953

2) Application of GSET: Predicting differences in genome size between males and females

- **Experimental results of 7 male and 7 female genomes:**

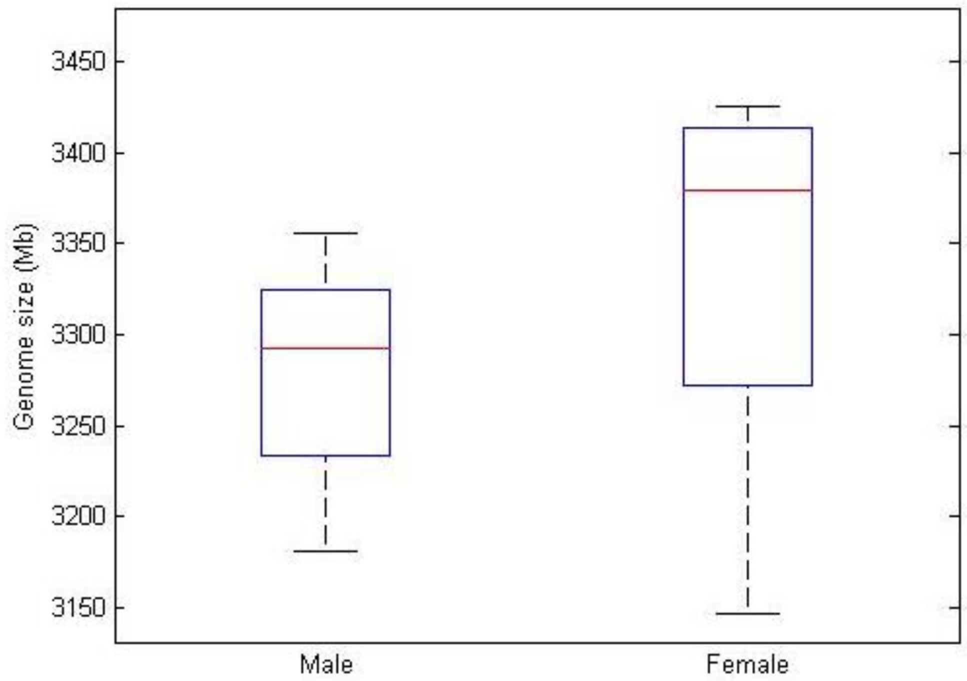

Fig. S2 Genome size estimation for male and female by GEST

- **Datasets information:**

Male: ERR1347682, ERR1347702, ERR1347706, ERR1347728, ERR1347738, ERR1395547, ERR1395570

Female: ERR1347657, ERR1347661, ERR1347662, ERR1347672, ERR1347679, ERR1347707, ERR1419089

Seven male and seven female sequencing datasets are used in this study. All datasets are downloaded from NCBI and estimated the genome size by GEST with k=21.

- **Experimental results:**

The average genome size of male and female in the experiments is 3279 Mb and 3428 Mb respectively. This conclusion is consistent with the fact: The female genome contains two X chromosomes, and male genome contains one X chromosome and one Y chromosome. Therefore, the female genome size is a bit bigger than that of male (Fig. S2).
